# *OpenPVSignal*: Advancing Information Search, Sharing and Reuse on Pharmacovigilance Signals via FAIR Principles and Semantic Web Technologies

**DOI:** 10.3389/fphar.2018.00609

**Published:** 2018-06-26

**Authors:** Pantelis Natsiavas, Richard D. Boyce, Marie-Christine Jaulent, Vassilis Koutkias

**Affiliations:** ^1^Centre for Research & Technology Hellas, Institute of Applied Biosciences, Thessaloniki, Greece; ^2^Lab of Computing, Medical Informatics & Biomedical Imaging Technologies, Department of Medicine, Aristotle University of Thessaloniki, Thessaloniki, Greece; ^3^Department of Biomedical Informatics, School of Medicine, University of Pittsburgh, Pittsburgh, PA, United States; ^4^Institut National de la Santé et de la Recherche Médicale, U1142, LIMICS, Paris, France; ^5^Sorbonne Universités, UPMC Univ Paris 06, UMR_S 1142, LIMICS, Paris, France; ^6^Université Paris 13, Sorbonne Paris Cité, UMR_S 1142, LIMICS, Villetaneuse, France

**Keywords:** drug safety, pharmacovigilance signals, adverse drug reactions, linked data, semantic web, ontologies, knowledge engineering, FAIR principles

## Abstract

Signal detection and management is a key activity in pharmacovigilance (PV). When a new PV signal is identified, the respective information is publicly communicated in the form of periodic newsletters or reports by organizations that monitor and investigate PV-related information (such as the World Health Organization and national PV centers). However, this type of communication does not allow for systematic access, discovery and explicit data interlinking and, therefore, does not facilitate automated data sharing and reuse. In this paper, we present *OpenPVSignal*, a novel ontology aiming to support the semantic enrichment and rigorous communication of PV signal information in a systematic way, focusing on two key aspects: (a) publishing signal information according to the FAIR (Findable, Accessible, Interoperable, and Re-usable) data principles, and (b) exploiting automatic reasoning capabilities upon the interlinked PV signal report data. *OpenPVSignal* is developed as a reusable, extendable and machine-understandable model based on Semantic Web standards/recommendations. In particular, it can be used to model PV signal report data focusing on: (a) heterogeneous data interlinking, (b) semantic and syntactic interoperability, (c) provenance tracking and (d) knowledge expressiveness. *OpenPVSignal* is built upon widely-accepted semantic models, namely, the provenance ontology (PROV-O), the Micropublications semantic model, the Web Annotation Data Model (WADM), the Ontology of Adverse Events (OAE) and the Time ontology. To this end, we describe the design of *OpenPVSignal* and demonstrate its applicability as well as the reasoning capabilities enabled by its use. We also provide an evaluation of the model against the FAIR data principles. The applicability of *OpenPVSignal* is demonstrated by using PV signal information published in: (a) the World Health Organization's Pharmaceuticals Newsletter, (b) the Netherlands Pharmacovigilance Centre Lareb Web site and (c) the U.S. Food and Drug Administration (FDA) Drug Safety Communications, also available on the FDA Web site.

## Introduction

### Definitions and problem statement

Pharmacovigilance (PV) is “*the science and activities related with the detection, assessment, understanding, and prevention of adverse effects or any other possible drug-related problems*” (World Health Organization, 2002). According to CIOMS (Council for International Organizations of Medical Sciences), a PV signal is “*information that arises from one or multiple sources (including observations and experiments), which suggests a new potentially causal association, or a new aspect of a known association, between an intervention and an event or set of related events, either adverse, or beneficial, that is judged to be of sufficient likelihood to justify verificatory action*” (Council for International Organizations of Medical Sciences (CIOMS), 2010). Adverse Drug Reactions (ADR) have significant consequences on public health, including a huge financial cost (Sultana et al., 2013), (Australian Commission on Safety and Quality in Health Care (ACSQHC), 2011). Therefore, facilitating the timely identification, early communication and the processing of a PV signal is imperative.

Typically, information regarding PV signals is disseminated via free-text reports. For example, the World Health Organization (WHO) releases its bi-monthly Pharmaceuticals Newsletter, containing a section devoted to PV signals identified and assessed by Uppsala Monitoring Centre^1^, while other organizations (e.g., the European Medicines Agency (EMA)^2^, the Food and Drug Administration (FDA) in the United States^3^, the Medicines and Healthcare products Regulatory Agency (MHRA) in the United Kingdom^4^, the Netherlands Pharmacovigilance Centre (Lareb)^5^) publish information regarding new PV signals on their Web sites. A typical structure of a PV signal report contains a title referring to the ADR and the respective drug(s), the author(s) of the report, a summary of the report and/or an introductory section, evidence supporting the signal (e.g., individual case safety reports (ICSRs), a.k.a. individual case reports or spontaneous reports, coming from Spontaneous Reporting Systems (SRS), and the literature), a conclusion and, finally, the respective bibliographic references.

Current free-text based dissemination practices do not facilitate automated processing, linkage and reuse of the respective information, since this information is not provided in a “computable” format, i.e., interoperable and well-structured format. The introduction of information technology (IT) tools and the use of semantically-enriched metadata can reinforce data expressiveness, exchange, linkage, and verification (through provenance information), as well as processing capabilities. The need for using metadata to annotate publicly available datasets has been pinpointed both by research and industry, and such technologies are currently used in order to facilitate data discovery and interlinking (Weaver and Tarjan, 2013; Noy, 2017). Such an improvement in PV signal dissemination could have significant impact for (a) PV experts exploiting such information to investigate candidate PV signals, (b) regulatory authorities which typically use such information to decide for further action on the specific drugs, and (c) healthcare professionals (HCPs) who may consult such reports during their clinical practice.

### Contribution and foundations of the current work

In this paper, we introduce *OpenPVSignal*, a novel ontology aiming to facilitate the publication of PV signal information in a reusable, extendable and computable knowledge representation format, thereby reinforcing access, discovery, and explicit data interlinking. We show how a semantically-enriched representation and communication of PV signals can be significantly facilitated through the Linked Data (Bizer, 2009) and the Semantic Web (Berners-Lee et al., 2001) paradigms. The ultimate goal of *OpenPVSignal* is the advancement of current practices as regards the publication and further processing of PV signal information by focusing on two key goals:

- publishing information following the FAIR (Findable, Accessible, Interoperable, and Reusable) data principles (Wilkinson et al., 2016), and
- exploiting automated reasoning capabilities upon the interlinked PV signal report data.

The term “Linked Data” refers to an ecosystem of technologies, recommendations and standards which aim at the interconnection of heterogeneous data in one unified processing realm (Heath and Bizer, 2011). The Semantic Web vision (Shadbolt et al., 2006) concerns the interconnection of semantic annotations of publicly available data through the Internet, and it is built upon Linked Data standards. The appropriateness of these paradigms/technologies to satisfy the main *OpenPVSignal* goals is summarized below:

- Linked Data (and therefore Semantic Web) standards and recommendations are based on the Resource Description Framework (RDF)^6^. RDF uses Uniform Resource Identifiers (URIs) to unambiguously identify resources (e.g., a Web page, a person, a data item, a process, a concept, etc.). URIs make data uniquely identifiable and, thus, *findable* and *accessible* through the Internet.
- RDF, RDF Schema^7^, and the Web Ontology Language (OWL)^8^—the main “languages” used to define knowledge in the Semantic Web paradigm—enable both *syntactic and semantic interoperability* by defining the rules for communicating data, the semantic structures to represent knowledge, and the interlinking of data with third-party datasets or ontologies.
- The use of existing semantic models facilitates *reusability* of the published data, as these are accompanied by well-defined metadata (e.g., about data provenance, time-related information, etc.). The adoption of these models facilitates their integration in already established processing pipelines based on the semantics of the referenced models.
- Finally, RDF Schema and OWL provide the ability to define concepts as well as high-level, semantic relations between them, (e.g., hierarchies among concepts defined as *classes, data and object properties, cardinality restrictions* on object properties, etc.). These are based on robust logical foundations [e.g., OWL semantics are based on Description Logics (Baader et al., 2004)] and, therefore, can be used by software (so-called “reasoners”) enabling automatic inference.

In order to semantically annotate PV signal information in compliance with the FAIR data principles, *OpenPVSignal* reuses well-known semantic models (described in detail in section *OpenPVSignal* Design). These models provide the means for (a) advanced knowledge expressiveness, (b) tracking provenance information, (c) automatic reasoning, and (d) semantic interoperability.

**Figure 1:**
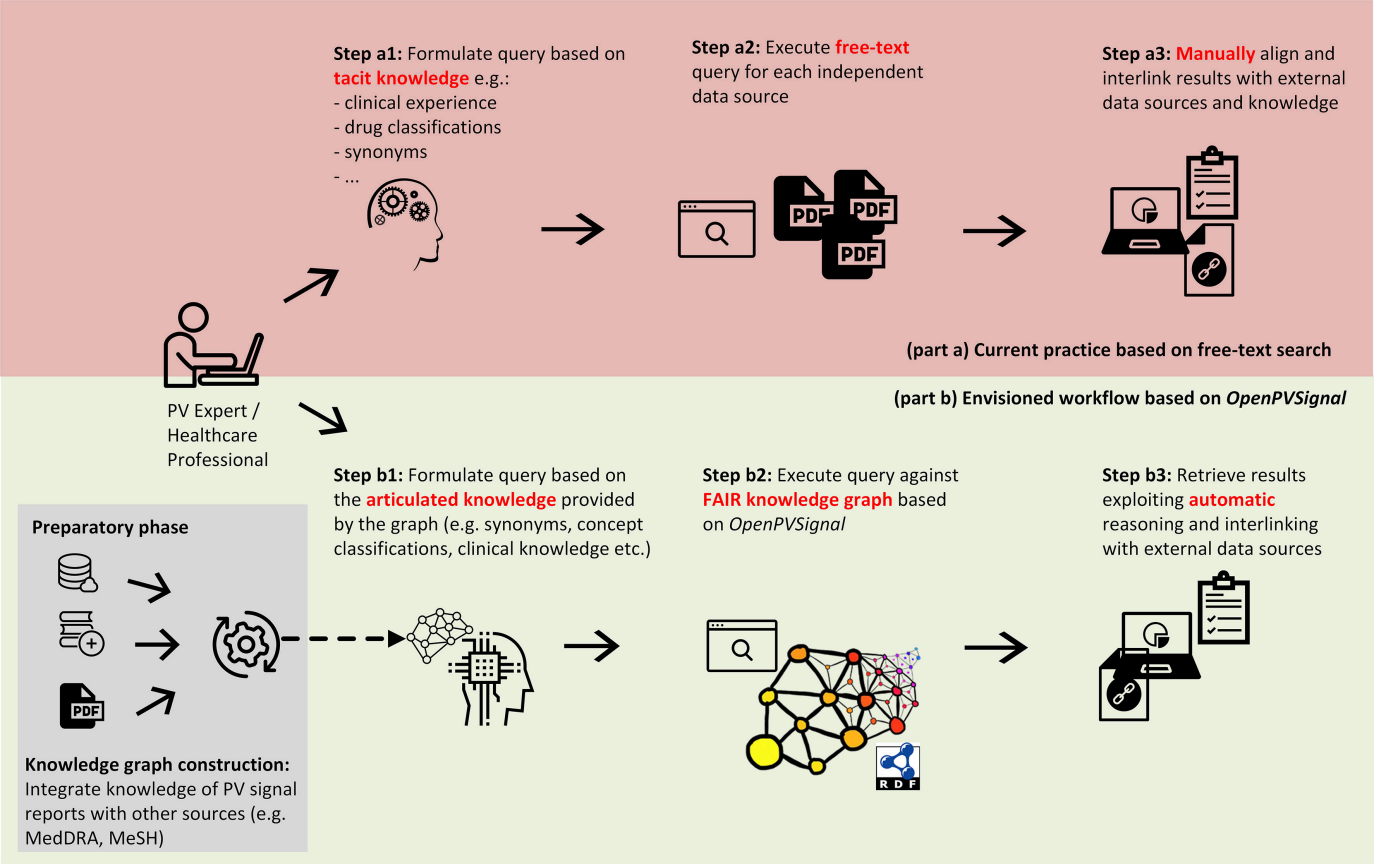
Comparing the current practice for searching PV signal information **(Top)** with the use of a FAIR knowledge graph based on *OpenPVSignal* **(Bottom)**.

The use of *OpenPVSignal* requires its instantiation for each PV signal report, i.e., representing the reports' content via the concepts of the *OpenPVSignal* ontology. Figure 1 depicts an information processing workflow using *OpenPVSignal* (part b), compared with the current typical approach followed during the search for PV signal information (part a). Typically, a PV expert or a HCP looking for PV signal information would manually conduct a search in the free-text resources provided by the respective PV organizations and manually aggregate the information of interest, based on his/her tacit knowledge and personal experience. This procedure is time consuming, possibly error-prone and heavily dependent on the specific end-user IT skills, as it involves multiple manual steps (Figure 1: steps a1, a2, and a3). The envisioned PV signal information processing workflow using *OpenPVSignal* allows the end-user to query a knowledge graph that meets FAIR principles (steps b1, b2, and b3), that will be built and processed (e.g., queried or modified) using a software application stack^9^. The knowledge graph creation includes the instantiation of the *OpenPVSignal* model using the free-text PV signal information and interlinking the obtained information with available knowledge sources, e.g., the Medical Dictionary for Regulatory Activities (MedDRA)^10^, Medical Subject Headings (MeSH)^11^, etc. This graph-based articulated knowledge significantly enhances the capabilities of linking, sharing, and automatically processing the original PV signal information. In the scope of this work, we illustrate the applicability and the added value of *OpenPVSignal* based on its instantiation for three PV signal reports, published by different organizations.

The structure of the paper is summarized as follows: Section “Related Work: ADR Representation Formalisms and Frameworks” presents related work regarding ADR representation formalisms, ontologies developed to define PV domain concepts and Linked Data knowledge sources for PV use cases. Section “*OpenPVSignal* Design” presents the key design decisions and the conceptual structure of *OpenPVSignal*. Section “Exemplar Application of *OpenPVSignal*” presents the application of *OpenPVSignal* on three signal reports published by different organizations, and its evaluation against the FAIR data principles. Finally, in section “Discussion” the main conclusions of the presented work are discussed, including future work directions.

## Related work: ADR representation formalisms and frameworks

Representation formalisms concerning ADRs have been employed/proposed in various studies, as well as Linked Data models and ontologies with a focus on PV.

For example, the Observational Health Data Sciences and Informatics collaborative (OHDSI) developed an evidence base that links evidence items (e.g., MEDLINE abstracts, drug product labels, spontaneous reports, etc.) to health outcomes of interest (Knowledge Base workgroup of the Observational Health Data Sciences Informatics (OHDSI) collaborative, 2017) using Web Annotation Data Model (WADM) graphs (Sanderson et al., 2017). Each graph represents drug and health outcome concepts mentioned in an evidence item as the *Body* of the annotation and the evidence item itself is summarized using metadata in the *Target* of the annotation. The concepts in the body of the annotation are mapped to the standard vocabulary used by the OHDSI collaborative^12^. This arrangement supports two use cases important to the collaborative: (1) to be able to quantify the evidence that supports a drug—health outcome of interest association, and (2) to enable users to review the context of the association in the original evidence sources. Investigators used the evidence base to develop machine learning algorithms that infer positive and negative drug—health outcome of interest associations (Voss et al., 2017).

ADEpedia (Jiang et al., 2013) encodes Adverse Drug Events (ADE) knowledge using a Linked Data serialization format exploiting several data sources (e.g., FDA Structured Product Labels (SPLs), reports from the FDA Adverse Event Reporting System (FAERS) and Electronic Medical Records). Biomedical ontologies, thesauri, and vocabularies, such as RxNorm^13^, NDF-RT^14^, and the Unified Medical Language System (UMLS)^15^, are used to specify concepts and normalize the interlinked data. The ADEpedia ontology consists of a rather lean concept schema, including two main concepts, namely, “Medication” and “ADE,” and does not include provenance information or statistical information on ADEs (Jiang et al., 2011).

OntoADR (Souvignet et al., 2016) is an OWL ontology, aiming to address the difficulties in expressing the inherent semantics of MedDRA in an OWL format, in order to support automatic reasoning via well-defined OWL semantics upon MedDRA terms. Similar to ADEpedia, OntoADR does not include statistical or provenance information regarding PV signals (Bousquet et al., 2014).

The Ontology of Adverse Events (OAE) aims to standardize and integrate medical adverse events (including ADRs), as well as to support computer-assisted reasoning (He et al., 2014). The two key OAE concepts are the *intervention* and the *adverse event*. OAE focuses on the semantic categorization of the interventions and the separation of them regarding causality. However, OAE is neither oriented toward provenance, nor on modeling information contained in free-text PV signal reports communicated by PV monitoring organizations.

Probably the most relevant ADR representation formalism compared to *OpenPVSignal* is the Adverse Event Reporting Ontology (AERO) (Courtot et al., 2014). AERO aims to support clinicians in the data entry phase, while reporting adverse events. It can also automate the classification of adverse event reports and improve the efficiency of discovering potential risks, with the ultimate goal to increase quality and accuracy of the reported information. However, AERO was not designed by taking into account the content of PV reports which are made publicly available by PV monitoring organizations and focuses on vaccine adverse effects (Adverse Events Following Immunization—AEFIs) via the application of a specific ADR signal analysis pipeline based on the Brighton guidelines. Apart from restricting its domain of application to vaccines and the specific ADR analysis workflow, AERO does not provide an explicit way to relate provenance or time-related information.

Compared to the above representation models, *OpenPVSignal* focuses on the representation of evidence-based PV signal information as communicated through the signal reports released by drug safety authorities. As mentioned in the “Introduction” section, these reports include supporting data originated from various sources, statistical measures (e.g., regarding disproportionality analysis of SRS data), as well as descriptions of the respective biochemical ADR mechanisms. Therefore, a dedicated ontology had to be defined, in order to leverage all these information types into one cohesive knowledge representation structure. Nevertheless, the above-mentioned models were studied in the scope of the current work concerning their concept definitions and their use of the Linked Data paradigm.

## *OpenPVSignal* design

*OpenPVSignal* was developed as an OWL ontology using the Protégé knowledge modeling tool (Musen and Protégé, 2015) (Protege, RRID:SCR_003299). The development of *OpenPVSignal* followed the NeOn knowledge engineering methodology (Suárez-Figueroa et al., 2012), applying the post-coordination approach (Stevens and Sattler, 2013) in an iterative fashion. Overall, we followed an application-driven approach by initially defining the concepts and the relations which served the intended use of the model, and then refining it in order to tackle issues that come-up during its real use.

*OpenPVSignal* reuses several existing ontologies, in order to exploit their semantics and to facilitate its adoption for other applications which rely on these models. In particular, we employed PROV-O, an ontology providing the formal concepts to represent and interchange provenance metadata independently of the application domain (Gil et al., 2013). In PROV-O, provenance is defined as “*information about entities, activities, and people involved in producing a piece of data or thing, which can be used to form assessments about its quality, reliability or trustworthiness*.” The PROV-O key concepts (i.e., *Entity, Agent*, and *Activity*) are defined as OWL classes, while relations (e.g., *wasAttributedTo*) are defined as OWL properties. The use of PROV-O in *OpenPVSignal* allows to clearly define provenance information for PV signal reports. For example, an indicative statement in the *OpenPVSignal* context would be that a signal report (instance of class *Entity*) is attributed to its author (instance of class *Agent*).

WADM is a semantic model enabling the annotation of Web content (e.g., Web pages, images, videos, documents, etc.) through the Linked Data paradigm (Sanderson et al., 2017). The main concepts of WADM are the *Annotation*, its *Target*, i.e., the annotated information (e.g., a video or a free-text document) and the annotation's *Body*, i.e., the information annotating the *Target*. In *OpenPVSignal*, WADM was used to annotate specific free-text snippets of PV signal reports. As an example, annotating a specific text snippet of a PV signal report referring to a drug action mechanism would require the definition of this snippet as the annotation's *Target* and the specific concept of the drug action mechanism as the annotation's *Body*.

Micropublications is a semantic model aiming to support (semi)automatic verification processes for data published in scientific articles (Clark et al., 2014). Micropublications also provide an OWL serialization that reuses PROV-O and the Open Annotation Core Data Model (WADM's predecessor). In Micropublications, a *Claim* is the main *Statement* argued, and each *Statement* or *Data* can be part of a *Claim*'s support or challenge graph. Using Micropublications in *OpenPVSignal*, a PV signal report conclusion is modeled as a *Claim*, while potential disproportionality analysis outcomes and the cited ICSRs are modeled as *Data*, and all free-text reporting elements are defined as subclasses of *ArticleText*.

Furthermore, OAE (He et al., 2014) was used in order to exploit the respective semantics and concept definitions. For example, the concept of *Drug Usage* was identified as equivalent to the “drug administration” concept defined in OAE. While OAE and *OpenPVSignal* have a different scope, they incorporate similar concepts. Therefore, the semantic interlinking of some key *OpenPVSignal* concepts with the respective OAE concepts enables their “understanding” by applications or knowledge models that have already adopted OAE semantics, further advancing the semantic interoperability of the *OpenPVSignal* model.

Finally, the semantics of “time to onset” information are described in *OpenPVSignal* using the *Duration Description* concept defined in the Time Ontology (TO). TO provides a vocabulary for expressing temporal concepts in OWL (Cox et al., 2017) and could be used to apply formal semantics and reasoning upon time-related information.

Moreover, *OpenPVSignal* enables the semantic enrichment of the respective data by allowing references to external terminologies and thesauri. For example, Anatomical Therapeutic Chemical Classification System (ATC)^16^, RxNorm, and DrugBank^17^ codes can be used to identify the respective drugs, International Statistical Classification of Diseases and Related Health Problems (ICD)^18^, Medical Subject Headings (MeSH) and SNOMED-CT^19^ codes can be used to identify diseases and MedDRA codes are used to identify adverse effects. This coded information is not necessarily present in the respective free-text signal reports. However, it can be easily retrieved from online services, facilitating this way the interlinking of source data with reference terminologies.

**Figure 2:**
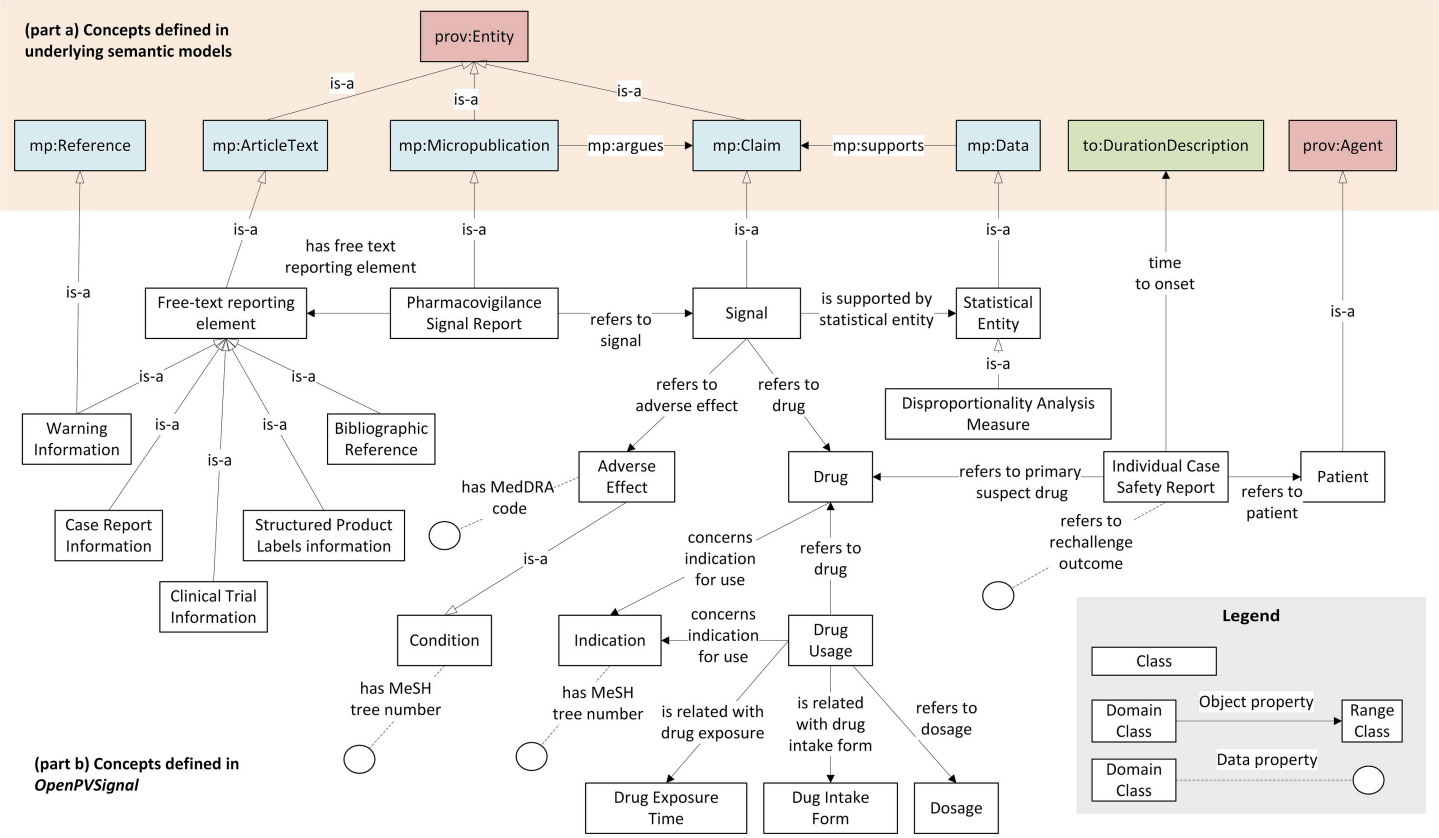
Main concepts defined in *OpenPVSignal* (part b), their reference to the concepts defined in the adopted semantic models e.g., Micropublications, Time Ontology, etc. (part a), and their hierarchical and interlinking relationships.

Figure 2 presents the main concepts of the *OpenPVSignal* model and their relations with the underlying semantic models, while Table 1 describes the main *OpenPVSignal* concepts and their relations. We refer to the concepts defined in the underlying semantic models by using the respective model abbreviations as a prefix in each concept name^20^, i.e., *mp* for Micropublications, *oae* for OAE, *prov* for PROV-O, and *to* for TO. For example, *mp:Claim* refers to the concept *Claim*, which is defined in the Micropublications semantic model. It should be noted that Figure 2 does not exhaustively depict all the concepts and relations of the model, in order to provide a comprehensive overview of the model and preserve readability. The full *OpenPVSignal* ontology is available through GitHub^21^ and its latest version can be downloaded using its fully dereferenceable base URI^22^. The complete documentation of the *OpenPVSignal* model is provided as Supplementary Material.

**Table 1:** The main concepts of *OpenPVSignal*.

**Concept**	**Description**
Pharmacovigilance signal report	Subclass of *mp:Micropublication* and, therefore, subclass of *prov:Entity*. Each free-text signal report corresponds to a *Pharmacovigilance Signal Report* instance.
Signal	Subclass of *mp:Claim* and equivalent to *oae:causal adverse event hypothesis* referring to the PV signal concept. Each instance of *Pharmacovigilance Signal Report* is related with one instance of the *Signal* class.
Free-text reporting element	Subclass of *mp:ArticleText* and consequently also subclass of *prov:Entity* corresponding to all the information that is being currently used to compile a human-readable PV signal report.
Drug	Subclass of *oae:processed material* representing the drug related information, including references to classification systems (e.g., in ATC). An instance of *Drug* is related with a *Signal*.
Drug class	Refers to the pharmacological class of the respective compound. For example, ibrutinib is an instance of *Drug* which “*belongs to class”* Protein Kinase Inhibitors, which is in turn an instance of the “*Drug Class”*.
Drug usage	Equivalent to *oae:drug administration* representing the reported usage of drugs, typically as part of an ICSR information.
Drug exposure time	Equivalent to *oae:exposure to toxic agent AE* representing the details of the patient's exposure to a specific drug in time. Instances of *Drug Usage* could refer to *Drug Exposure* instances, when such information is available.
Drug intake form	Concerns the form of the drug taken by the patient (e.g., injection, pill, etc.). Instances of *Drug Usage* could refer to *Drug intake form* instances, when such information is available.
Dosage	Represents information regarding the regulated administration of individual doses, the quantity of drug to be administered at one time, or the total quantity administered during a specified period, i.e., the dosage not only tells the quantity of medicine to be taken, but it also tells the frequency or the number of times a medicine has to be taken by the patient. Instances of *Drug Usage* would typically refer to *Dosage* instances.
Condition	Equivalent to *oae:disease* representing diseases or any kind of phenotypic information that could be of medical relevance, which is specified through ICD-10 codes.
Adverse Effect	A subclass/subconcept of *Condition*, equivalent to *oae:adverse effect* representing the adverse effects of drug administration. Instances of *Adverse Effect* are typically referred by a *Signal* instance.
Indication	Represents the reason that a drug is administered for. Its instances are typically referred by *Drug* instances.
Individual Case Safety Report	Subclass of *oae:drug adverse event reporting* and *mp:Data* representing ICSRs submitted in SRSs such as VigiBase, Lareb, or FAERS. Its instances refer to the specific reported case's details (e.g. “time to onset” information by pointing to *to:DurationDescription*).
Patient	Equivalent to *oae:patient* and subclass of *prov:Agent* representing basic patient information (e.g., age and sex). Such information is included in ICSRs and, therefore, an instance of *Individual Case Safety Report* refers to a *Patient* instance.
Statistical measure	Represents data that have a specific processing value, e.g., disproportionality analysis measures related to ICSRs.
Warning information	Subclass of *mp:Reference* and equivalent to *oae:contraindication* referring to already known contraindication information (e.g., warnings contained in SPLs).

## Exemplar application of *OpenPVSignal*

In this section, we present an exemplar application of *OpenPVSignal*, aiming to illustrate its applicability and validate its effectiveness by highlighting the added value of interlinkage and automatic reasoning. In particular, we describe the instantiation of *OpenPVSignal* based on three PV signal reports published by different organizations and also present two example use cases to demonstrate the value of data interlinking and automatic reasoning. Finally, we provide a validation of the model's compliance with the FAIR principles.

### Elaborated PV signal reports

The use of *OpenPVSignal* is demonstrated by elaborating on three specific types of PV signal reports: (a) signal information contained in the WHO Pharmaceuticals Newsletter, (b) reports from the Netherlands Pharmacovigilance Centre Lareb, and (c) announcements contained in the FDA Drug Safety Communication. The WHO Pharmaceuticals Newsletter disseminates “*information on the safety and efficacy of pharmaceutical products, based on information received from a network of “drug information officers” and other sources…*”^23^. Its “Signals” section presents specific PV signal information in a free-text format, also referring to relevant ICSRs contained in VigiBase^24^, publications, as well as other data sources. Lareb publishes signal reports also in free-text format via its Web site as soon as a signal is identified, which are searchable through a publicly available user interface^25^. Finally, FDA publishes Drug Safety Communication announcements as online reports, which are also publicly available through a Web site^26^. The respective PV signal information sources and formats have been selected as representative ones, due to the following reasons:

- *Credibility:* These reports originate from reference organizations and they are widely recognized by the PV community and HCPs worldwide. Furthermore, they are based on valid sources of information, properly curated by PV experts, therefore, providing reliable signal information.
- *Information richness and heterogeneity:* They contain a lot of information originated from diverse data sources like SRS, the literature, etc., along with references to the raw data, and also present information in different granularity levels. For example, Lareb PV signal reports refer to VigiBase ICSRs and Dutch case reports. In contrast, an FDA Drug Safety Announcement may not refer to ICSRs explicitly, but provides aggregated information. Furthermore, some PV signal reports include statistical figures concerning the specific signal (e.g., disproportionality analysis outcomes).

The PV signal reports selected for the example instantiation of the *OpenPVSignal* model refer to (a) the signal concerning *ibrutinib-induced pneumonitis*, published in the third WHO Pharmaceuticals Newsletter of 2017 (Pal and Tanaka, 2017), (b) the signal of *(es)omeprazole-induced tinnitus* published in 2013 by LAREB (Nederlands Bijwerkingen Centrum Lareb, 2013), and (c) the signal of Proton Pump Inhibitors (PPIs) leading to hypomagnesemia, communicated by FDA (FDA Center for Drug Evaluation Research, 2011).

### *OpenPVSignal* instantiation

**Figure 3:**
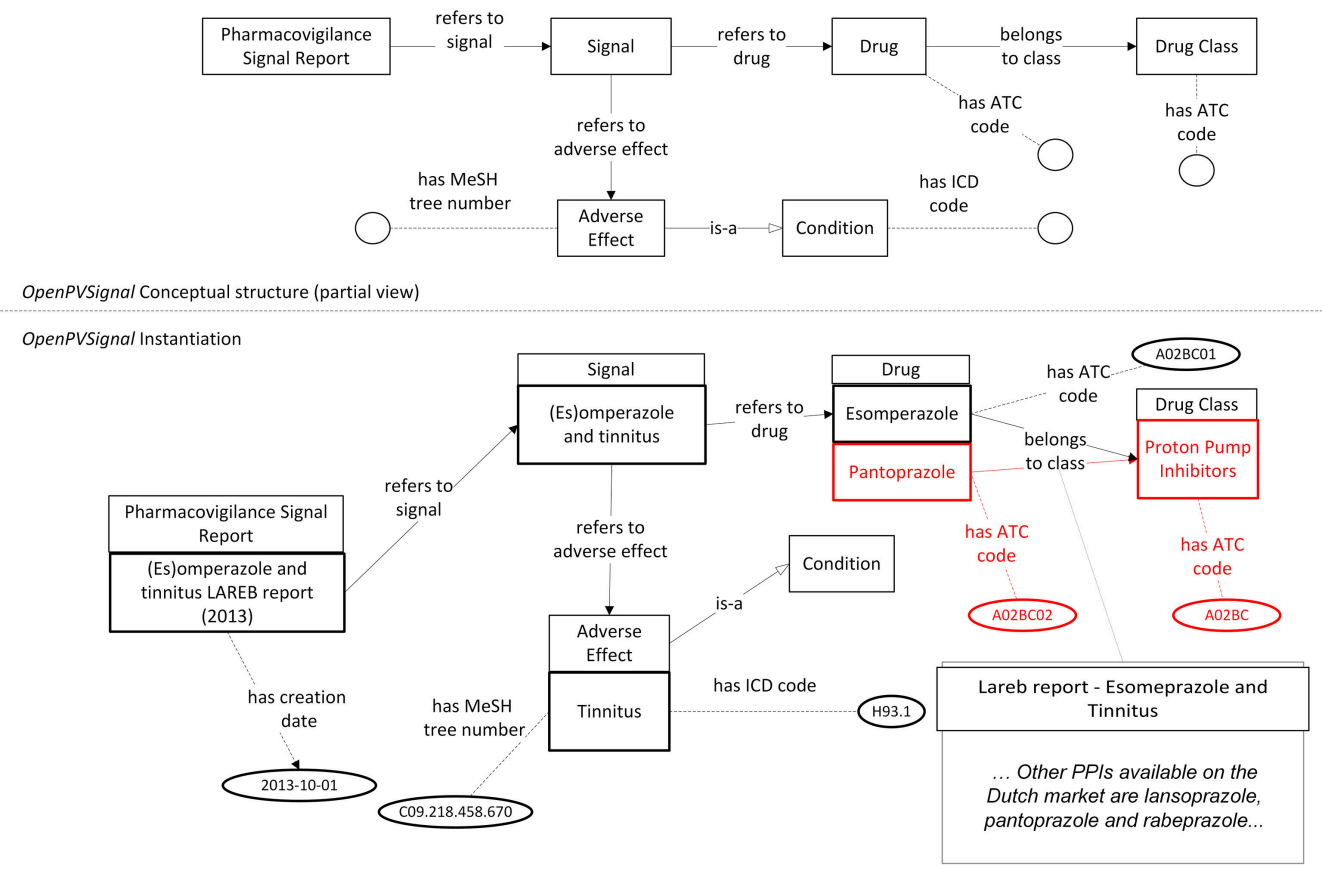
Main signal information contained in the Lareb PV signal report modeled using *OpenPVSignal*. The respective *OpenPVSignal* conceptual structure is depicted in the upper part and thick outlines denote instances of *OpenPVSignal* classes in the bottom part of the figure. The identification of Pantoprazole as a drug belonging to the Proton Pump Inhibitors class and the specific ATC codes are highlighted in red. The respective free text from where this relation has been extracted, is depicted in the bottom right corner of the figure.

**Figure 4:**
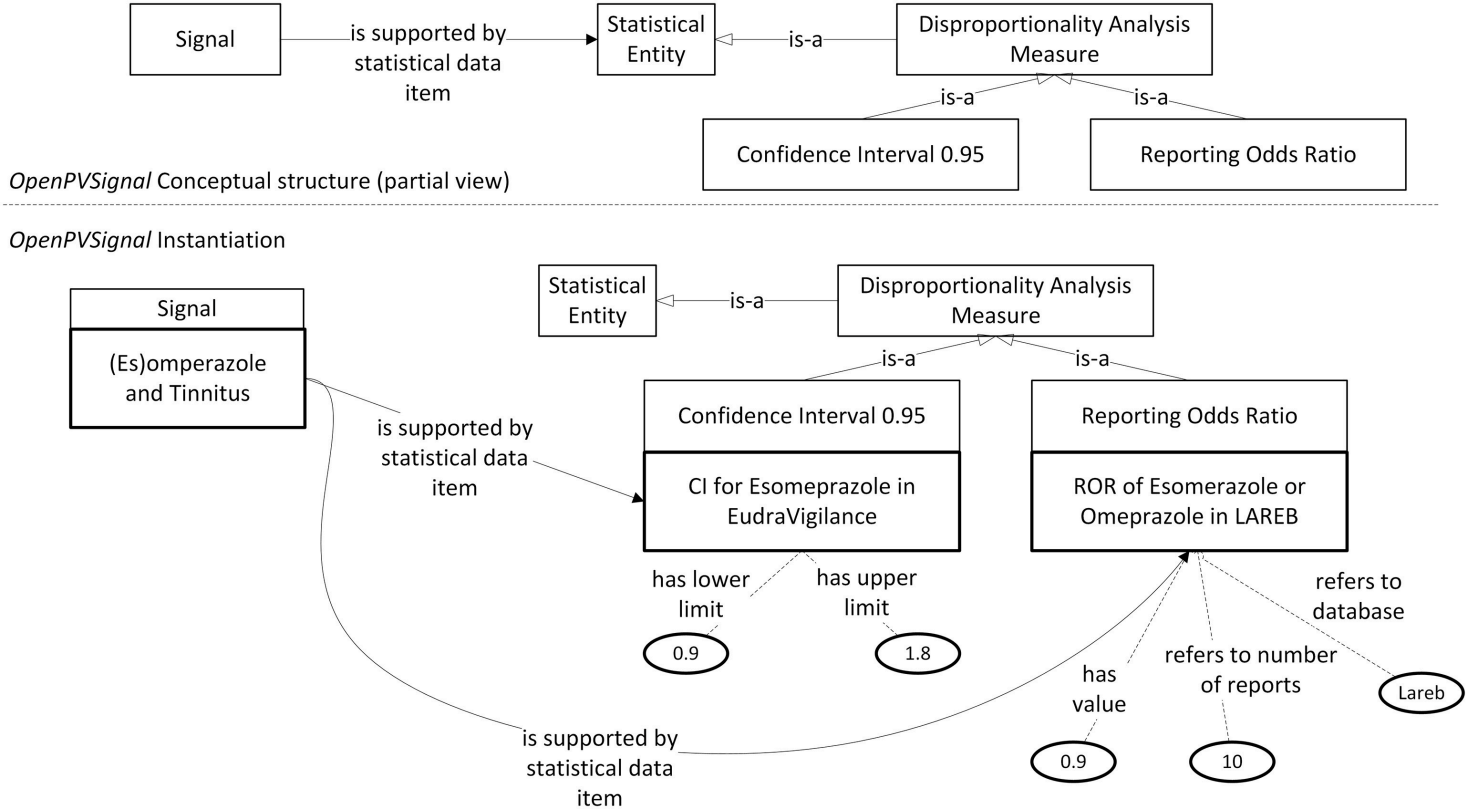
Disproportionality analysis outcomes contained in the Lareb report expressed via *OpenPVSignal*. The respective *OpenPVSignal* conceptual structure is depicted in the upper part and thick outlines denote instances of *OpenPVSignal* classes in the bottom part of the figure.

Currently, there is no automatic tool for instantiating *OpenPVSignal* from the original data sources. Therefore, the instantiation of the elaborated PV signal reports has been performed manually, using Protégé 5.2. Figures 3, 4 (partially) depict the *OpenPVSignal* modeling of information contained in the Lareb report. The upper part of each figure depicts the respective *OpenPVSignal* conceptual model part. The respective instantiations referring to the specific signal information are shown at the bottom of each figure, highlighting instances of the respective *OpenPVSignal* concepts as thick rectangles. It should be noted that we present an overview rather than a detailed walkthrough of the *OpenPVSignal* instantiation process, as the detailed example instantiations are publicly available in the *OpenPVSignal* page in GitHub^27^.

As shown in Figure 3, apart from the main signal information (i.e., the drug and the adverse effect), complementary information can also be modeled through *OpenPVSignal*. For example, the similar effects of other drugs that belong to the PPIs class are also elaborated in the Lareb report and specific drugs with the same or similar effects are mentioned, e.g., pantoprazole highlighted in Figure 3 with red color. The free-text snippet of the report from which this specific information is inferred is also depicted in the bottom-right corner of Figure 3. Figure 4 depicts the modeling of the disproportionality analysis outcomes mentioned in the Lareb PV signal report.

**Figure 5:**
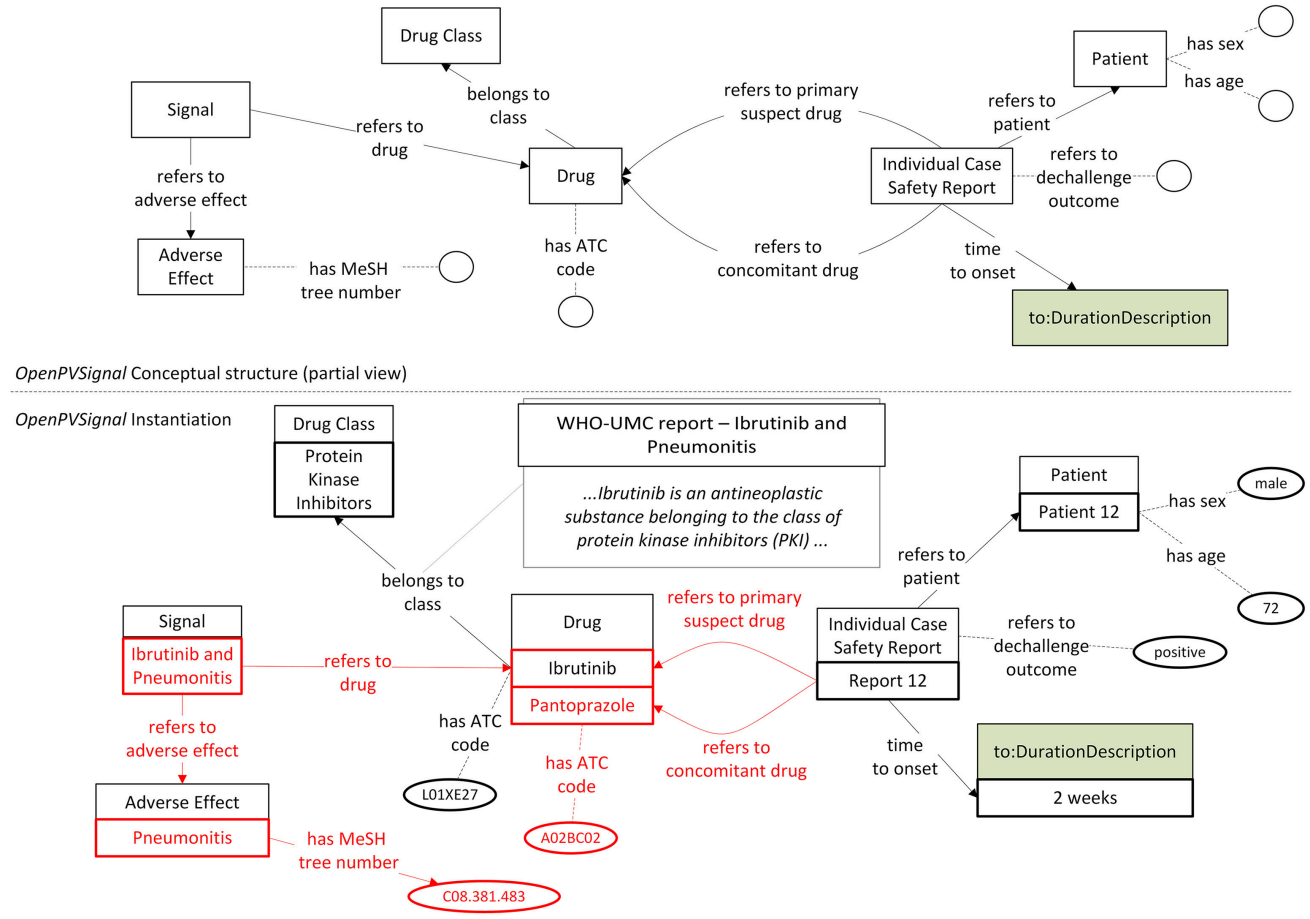
VigiBase ICSR data contained in the WHO Pharmaceuticals Newsletter PV signal expressed via *OpenPVSignal*. The reference of the specific ICSR to Pantoprazole, the related instances and the respective MeSH and ATC codes are highlighted in red.

The instantiation of the signal report selected from the WHO Pharmaceuticals Newsletter is partially depicted in Figure 5. Besides the drug and the adverse effect, Figure 5 depicts also the modeling of ICSRs mentioned in the respective PV signal report, i.e., information referring to ICSR with ID 12, as well as the “time to onset” information expressed using concepts defined in TO. It should be noted that while ICSR 12 refers to the ibrutinib-pneumonitis signal, it also refers to pantoprazole as a concomitant drug in an ICSR referring to pneumonitis (conceptual linking between pantoprazole and pneumonitis is highlighted with red color). The information depicted in Figure 5 can be of clinical relevance for the investigation of a potential PV signal as both time information and concomitant drugs can be considered in the causality analysis between a drug and the adverse effect.

### Added value of data interlinking and reasoning via *OpenPVSignal*

**Figure 6:**
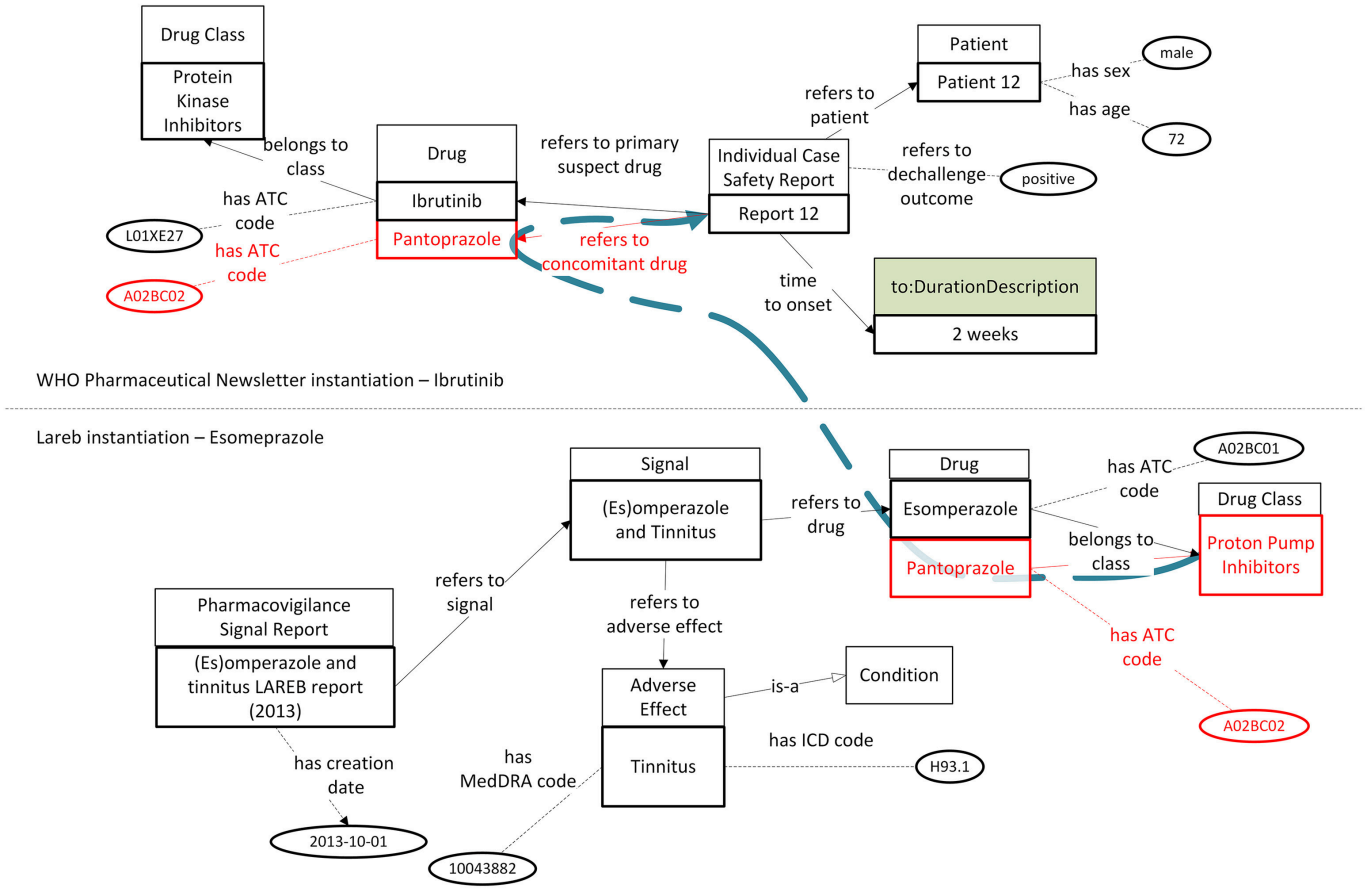
Interlinking and reasoning upon PV signal reports from the WHO Pharmaceuticals Newsletter and Lareb (the blue dashed line depicts the logical path interlinking PPIs with ICSR 12 in the WHO Pharmaceuticals Newsletter).

In order to highlight the value of *OpenPVSignal*, we present two example use cases according to which a user exploits the *OpenPVSignal* instantiations presented above. In the first use case, we assume that the user investigates new, possible adverse effects of drugs belonging to the PPI class. Typically, she/he would search the free-text PV signal information sources, in order to find signals referring to PPIs. While the considered Lareb PV signal report and the FDA Drug Safety Communication explicitly refer to PPIs as the class of drug pantoprazole, the WHO Pharmaceuticals Newsletter does not refer to PPIs, since ibrutinib does not belong to the PPI class. Therefore, although the expert would have identified the two PV signal reports (by LAREB and FDA) through manual search, probably she/he would not notice ICSR 12 (depicted in Figure 5) mentioned in the WHO Pharmaceuticals Newsletter. In this ICSR pantoprazole (which belongs to the PPI class) is referred as a concomitant drug in a PV signal report concerning another drug, irrelevant with the PPI class. The use of a knowledge graph based on the *OpenPVSignal* model enables the retrieval of ICSR 12 as relevant with the requested PPI signal information. In Figure 6, the parts highlighted in red provide the interlinking between the PPI drug class and the ICSR mentioned in the WHO Pharmaceuticals Newsletter.

**Figure 7:**
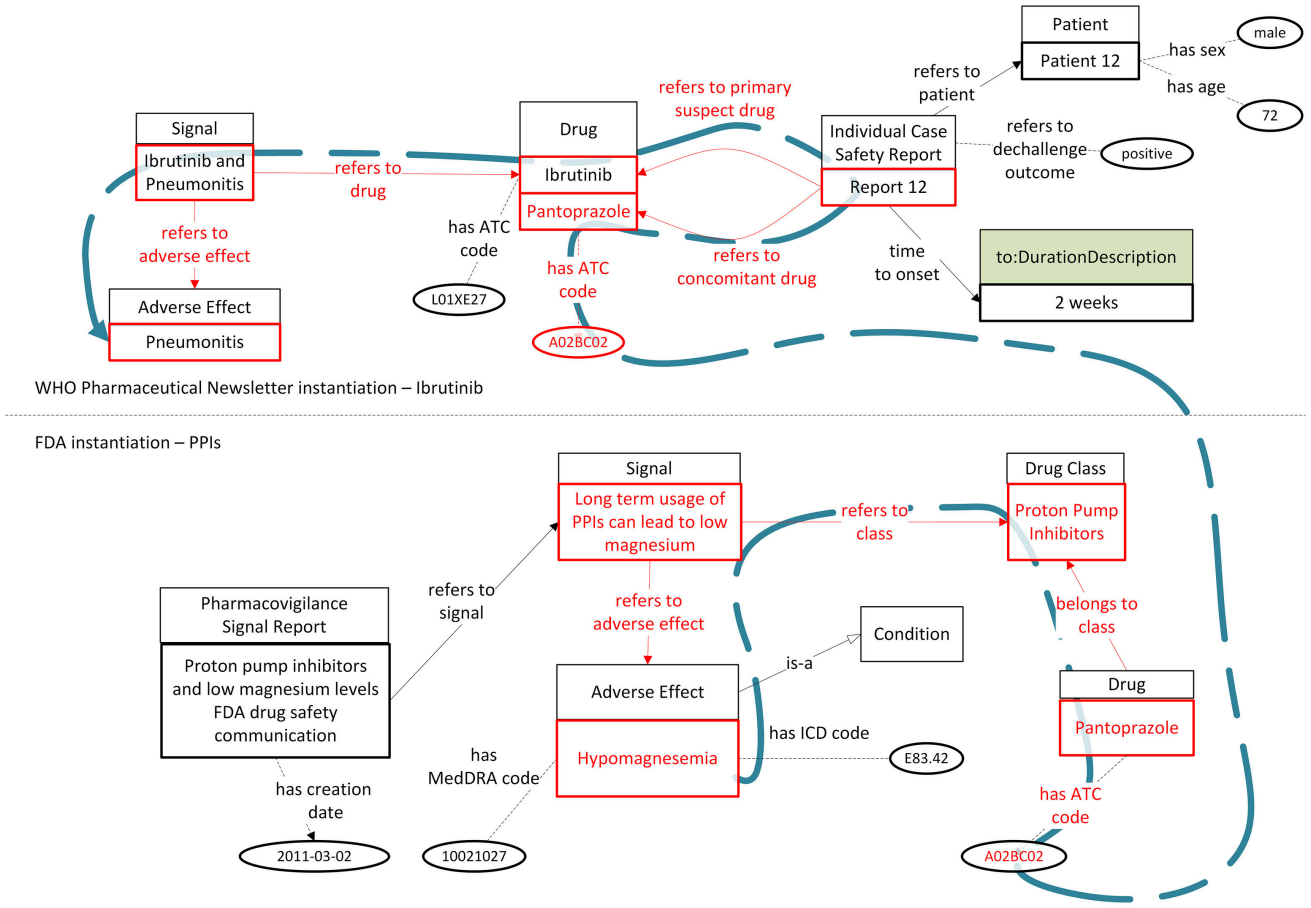
Interlinking and reasoning upon PV signal reports from the WHO Pharmaceuticals Newsletter and the FDA Drug Safety Communications (the blue dashed line depicts the logical path interlinking hypomagnesemia and pneumonitis with ICSR 12 mentioned in the WHO Pharmaceuticals Newsletter).

As a second use case, let us assume that an expert reads a scientific article claiming that mice tests indicate a relation between magnesium deficiency and pneumonitis (Nasulewicz et al., 2004). In order to investigate this claim, she/he searches in the considered PV signal sources for drugs which are related with magnesium deficiency and are also reported to be related with pneumonia. While the FDA Drug Safety Communication indicates a relationship between PPIs and magnesium deficiency and the WHO Pharmaceuticals Newsletter refers to the use of pantoprazole as a concomitant drug in an ICSR regarding pneumonia, this information could only be retrieved if the expert expanded her/his free-text search to include all drugs belonging to PPIs too, thus, including pantoprazole as a search keyword. However, using a knowledge graph based on the *OpenPVSignal* model enables the retrieval of ICSR 12 as relevant with hypomagnesemia and pneumonia. As depicted in Figure 7, the parts highlighted in red can provide the interlinking between the concept of magnesium deficiency, the PPI drug class, and the specific ICSR mentioned in the WHO Pharmaceuticals Newsletter.

While the data interlinking presented in these two exemplar use cases can be important for a drug safety investigation, it is difficult to be identified manually, as it would require a significant expansion of the search space across distinct, multiple data sources. This expansion would complicate the investigation process and proliferate the chance of missing useful information. Therefore, the use of the Linked Data paradigm as employed in *OpenPVSignal* can be particularly helpful to avoid missing information that may be useful when searching for PV signal information across multiple report sources. It should be noted that the logical paths depicted using the blue dashed line are automatically inferred by reasoners, despite the fact that these relationships are not explicitly declared in the respective instantiations.

### *OpenPVSignal* evaluation: compliance with the FAIR principles

The four main guiding principles of the FAIR model, namely, Findable, Accessible, Interoperable, and Reusable, are further analyzed in a set of 15 more detailed guidelines presented in Table 2 (Wilkinson et al., 2016). Applying FAIR guiding principles to PV data would inherently enhance their value and, therefore, enhancing the “FAIRness” of such data is one of main goals of *OpenPVSignal*. Therefore, in Table 2 we present a qualitative evaluation of *OpenPVSignal* against the FAIR principles. It should be noted that in the context of the analysis presented in Table 2, we consider that the term *data* refers to the original free-text PV signal reports and the term *metadata* refers to the respective *OpenPVSignal* instantiations.

**Table 2:** The FAIR Guiding Principles and *OpenPVSignal* approach compliance.

**FAIR principles**		**Evaluation of *OpenPVSignal* approach**
**TO BE FINDABLE:**		
F1	(meta)data are assigned a globally unique and eternally persistent identifier.	*OpenPVSignal* is based on OWL and RDF which identify each resource (e.g., instance of a concept or the concepts per se) through an Internationalized Resource Identifier (IRI). IRIs corresponding to the specific instantiations of *OpenPVSignal* are based on Internet URLs and, therefore, the identifiers used are globally unique and persistent. For example, the base URI of the use case instantiation elaborated for the Lareb PV signal report corresponds to a URL^*a*^. These URLs are provided by purl.org and currently redirect to our GitHub URL where the respective OWL files are hosted. In case of moving the specific OWL files to another URL, the redirect rule in purl.org would change, keeping the public URL of the files constant.
F2	Data are described with rich metadata.	This is a subjective requirement as it heavily depends on the definition of “richness”. *OpenPVSignal* defines the concepts required for the intended use cases, namely, the interlinking and the further processing of PV signal reports. The instantiations (metadata) presented in section “Added Value of Data Interlinking and Reasoning” depict the richness of the model in the context of the elaborated PV use cases. However, the development of such a model is a continuous and iterative work. Therefore, we expect that the *OpenPVSignal* conceptual model will be further developed and refined, contributing to a richer and more accurate metadata model.
F3	(meta)data are registered or indexed in a searchable resource.	*OpenPVSignal* aims at facilitating and enhancing the process of publishing PV signal report data, which are available online. Therefore, we consider that the *OpenPVSignal* instantiations would also be published online and could be easily retrieved through typical Internet search. However, it should be clarified that this issue is not related with the presented model per se, but with the applications that will use it. Therefore, indexing the produced *OpenPVSignal* instantiations is considered an application-level issue.
F4	metadata specify the data identifier.	*OpenPVSignal* instantiations are directly linked to original data, i.e., the free-text PV signal reports. The original free-text report public URL is considered as its public identifier (reference to the original report is done using the *rdf:isDefinedBy* annotation property).
**TO BE ACCESSIBLE:**		
A1	(meta)data are retrievable by their identifier using a standardized communications protocol.	We assume that the instantiations of *OpenPVSignal* would use fully dereferenceable URLs as identifiers. In that case, relevant Internet protocols (i.e., HTTP and IP) can be considered as *OpenPVSignal*'s lower-layer communication protocol. The presented instantiations have persistent URLs as identifiers^*a,b,c*^ and they can be directly retrieved through them, using a simple HTTP request.
A1.1	The protocol is open, free, and universally implementable.	The Internet is the *OpenPVSignal*'s underlying communication protocol which is inherently open, free and universally implementable.
A1.2	the protocol allows for an authentication and authorization procedure, where necessary.	This requirement is satisfied using normal Internet security measures, since *OpenPVSignal* instantiations are accessible through the Internet.
A2	metadata are accessible, even when the data are no longer available.	In the context of *OpenPVSignal* intended use, even if an original PV report becomes unavailable, the respective *OpenPVSignal* instantiation could remain online as it is a completely independent OWL file.
**TO BE INTEROPERABLE:**		
I1	(meta)data use a formal, accessible, shared, and broadly applicable language for knowledge representation.	The structure of the *OpenPVSignal* model is defined based on OWL and RDF which are open and widely accepted standards, independent of specific platforms (e.g., operating systems, programming languages etc.) and, thus, universally implementable. *OpenPVSignal* instantiations are compiled in OWL, therefore satisfying the specific requirement.
I2	(meta)data use vocabularies that follow FAIR principles.	The underlying semantic models of *OpenPVSignal*, namely PROV-O, Micropublications, WADM, OAE and TO, are compliant with the FAIR principles and their overall rationale. Furthermore, the vocabularies/terminologies that are referenced in *OpenPVSignal* (i.e., ICD-10, ATC, MeSH and MedDRA) are compliant with the specific requirement. The use of licensed vocabularies (e.g. MedDRA) does not affect the “FAIRness” of the overall conceptual model, as FAIR principles do not require a “free-of-charge” model. More specifically, principle R1.1 requires that “*(meta) data are released with a clear and accessible data usage license*” without requiring this license to be free of charge.
I3	(meta)data include qualified references to other (meta)data.	*OpenPVSignal* references to a set of underlying semantic models, which are fully qualified as they are based on the RDF and OWL referencing mechanisms and refer to their respective RDF implementations.
**TO BE RE-USABLE:**		
R1	meta(data) have a plurality of accurate and relevant attributes.	The *OpenPVSignal* model incorporates several concepts and attributes from the domain of PV signal communication. The ones presented in the manuscript are only indicative, while full details are provided in our GitHub repository dedicated to *OpenPVSignal*.
R1.1	(meta)data are released with a clear and accessible data usage license.	The data currently investigated are publicly available online and the *OpenPVSignal* model is provided as an open source implementation, under GNU license, including its exemplar instantiations. However, the license of the *OpenPVSignal* instantiations produced by a specific application are left to be decided on the application level.
R1.2	(meta)data are associated with their provenance.	*OpenPVSignal* utilizes Micropublications and PROV-O to enhance provenance tracking capabilities of the PV signal reports' data. As demonstrated in Figure 3 and Figure 5, the produced RDF statements can be directly associated with the respective free-text snippet in the original free-text PV signal report by using WADM.
R1.3	(meta)data meet domain-relevant community standards.	There are currently no domain-relevant community standards regarding the publication of PV signal reports. However, in this paper we demonstrate the usage of *OpenPVSignal* upon three different PV signal report formats (WHO Pharmaceuticals Newsletter, Lareb reports and FDA Drug Safety Communications), which could be considered indicative in publishing PV signal reports. Furthermore, we semantically related *OpenPVSignal* concepts with concepts defined in OAE (He et al., 2014).

a http://purl.org/OpenPVSignal/examples/Lareb_2013_3_Esomeprazole_and_tinnitus.owl

b http://purl.org/OpenPVSignal/examples/WHO_UMC_Pharmaceuticals_Newsletter_2017_3_Ibrutinib_and_pneumonitis.owl

c http://purl.org/OpenPVSignal/examples/FDA_Drug_Safety_Communication_2_3_2011_PPIs_and_low_magnesium_levels.owl

Furthermore, we have evaluated our proposed model against the respective emerging FAIR metrics framework^28^ proposed by the FAIR Metrics Group^29^. As the current version of the proposed metrics refers to rather low-level technical details, we consider this evaluation process out of scope for the journal audience, and we consider our qualitative analysis presented in Table 2 more suitable in order to illustrate the “FAIRness” of *OpenPVSignal*.

Based on the presented analysis, we can conclude that *OpenPVSignal* complies fully with the FAIR principles.

## Discussion

The value of Linked Data and Semantic Web technologies for pharmacological research has been illustrated in various studies and projects. Beyond research on ADR representation, which was extensively presented in section “Related Work: ADR Representation Formalisms and Frameworks”, of note is the Linked Open Drug Data (LODD) initiative (Samwald et al., 2011), a project conducted by the W3C Semantic Web for Health Care and Life Sciences Interest Group (HCLS IG), exploiting semantic discovery techniques to automatically interlink diverse datasets. The Bio2RDF project transforms a variety of life science data sources to RDF [among which DrugBank (Law et al., 2014), SIDER (Kuhn et al., 2016) and FDA Structured Product Labels (Hassanzadeh et al., 2013)], through a well-defined transformation process. Furthermore, OpenPHACTS is an ongoing European initiative, building the so-called “Open Pharmacological Data Space” by collecting and integrating biochemical data from several heterogeneous sources (Hu and Bajorath, 2014), aiming to facilitate the discovery of new drugs.

Interlinking heterogeneous datasets to facilitate drug research using Linked Data has been presented in Boyce et al. (2014), in the scope of the OHDSI initiative, elaborating also on PV use cases [Knowledge Base workgroup of the Observational Health Data Sciences Informatics (OHDSI) collaborative, 2017], while an approach for combining the results of diverse computational PV signal detection methods applied in diverse data sources using Semantic Web technologies was elaborated in the SAFER project (Koutkias and Jaulent, 2015). The PredicTox project aimed to foster ADR prediction through the combination of various data sources (Zaman et al., 2017). Interestingly, biomedical knowledge sources have also been integrated and used for drug repurposing (Himmelstein et al., 2017).

A Linked Data model targeting PV signal investigation was presented in Natsiavas et al. (2017). This model was partially based on RDF resources available via Bio2RDF (Callahan et al., 2013), namely, DrugBank, SIDER, Linked SPL, PharmGKB, and ClinicalTrials.gov, and it was evaluated using three reference datasets containing both positive and negative PV signal controls. The evaluation process confirms or rejects each candidate PV signal based on the information provided by the model. The result was compared for the three reference datasets, aiming to highlight the value of interlinking various data sources for PV signal investigation.

In the current work, we aimed to address the shortcoming arising from the free-text format based on which PV signal reports are made publicly available from organizations which monitor and investigate PV signals. This practice does not facilitate systematic search and automatic interlinking of information. To this end, we presented *OpenPVSignal*, a novel ontology which provides the knowledge model and the semantics upon which PV signal information contained in the current reports could be annotated and enriched. Through the adoption of the Linked Data paradigm and Semantic Web standards, *OpenPVSignal* enables overcoming the diversity in the provided free-text report's syntactic structure and the provided information granularity level. Based on common practices in ontology modeling, *OpenPVSignal* reuses several existing semantic models, namely, Micropublications, PROV-O, WADM, OAE, and TO. For illustration purposes, three PV signal reports originated from different sources were instantiated and two exemplar use cases exploiting these instantiations highlighted the value of *OpenPVSignal*.

Data interlinking, retrieval, and automatic reasoning are crucial in the PV domain, where the currently applied typical workflow for signal generation and verification relies on complex manual exploration of multiple (mostly free-text) data sources (Koutkias et al., 2017). The advantages of using *OpenPVSignal* could be summarized as follows:

1. Facilitates the reusability of valuable information, which can be currently lost due to its unstructured nature.
2. Saves time and effort, as it significantly facilitates the automation of manually conducted work.
3. Provides the basis for an advanced computational framework, aiming to facilitate PV signal assessment by interlinking the respective information with other data sources and by applying semantic reasoning.
4. Given that the RDF representation uses URIs to uniquely identify each information resource (e.g., an ICSR), the use of Linked Data and *OpenPVSignal* could facilitate the detection of duplicate information and, therefore, allow for their better processing.

The limitations of the presented work include: (a) the need for a more thorough validation of *OpenPVSignal* by elaborating on PV signal reports published by additional sources and, consequently, (b) the need for potential extensions of our ontology model and (c) the size of the model, which can be a significant barrier for automatic reasoning^30^. The definition of the *OpenPVSignal* concepts followed the post-coordination ontology development approach, in a step-by-step fashion and according to their use in the free-text PV signal reports of the considered sources. Thus, these definitions were not a result of an exhaustive procedure, which could entail the analysis of PV signal reports that are published by all relevant organizations. Providing *OpenPVSignal* as an open-access resource and offering a transparent open-source development process is a key decision toward restricting the above limitations. Overall, we consider the development of the *OpenPVSignal* model an ongoing process, driven by its use in real-world applications.

It should be noted that the main use case of *OpenPVSignal* concerns the publication process of PV signal information that is already publicly available in free-text reports. The process of publishing PV signal information and the security risks or ethical issues related with this information are not relevant with the information representation format and as such, we consider them out of the scope of this work. Furthermore, it should be clarified that *OpenPVSignal* neither employs nor proposes specific statistical processing method(s) for signal detection. *OpenPVSignal* is a knowledge representation model for publishing PV signal information and this information may typically include references to the statistical methods/measures used for signal detection. Thus, *OpenPVSignal* provides the mechanism to encode this information without elaborating on its assessment.

Besides extending the *OpenPVSignal* validation, our future work concerns: (a) the development of a tool to facilitate the automatic population of *OpenPVSignal* with the content of already released PV signal reports by applying Natural Language Processing techniques, in order to construct the respective knowledge graph, (b) the development of a user-friendly tool to create, publish, browse and query *OpenPVSignal* instances, appropriate for use by PV signal monitoring organizations and drug regulatory authorities, and (c) the development of a knowledge-based, computational framework for assessing candidate PV signals by exploiting the semantic reasoning capabilities that *OpenPVSignal* offers. The development of such tools can be facilitated by frameworks that can automatically extract information from free-text data sources. For example, BioKB (Biryukov et al., 2017) provides a paradigm for semantically annotating free-text content and interlinking it with reference vocabularies, while PoeM (Gaignard et al., 2016) provides a way for extracting provenance information in a Linked Data format.

Overall, we believe that *OpenPVSignal* can be the basis for an advanced PV signal dissemination mechanism, appropriate for adoption by organizations who investigate and publish PV signal information and drug regulatory authorities.

## Author contributions

VK conceived and supervised the study. PN implemented the ontology model. All the authors contributed to the design and critical review of the ontology model as well as to the manuscript writing. All the authors reviewed and approved the content of the manuscript.

### Conflict of interest statement

The authors declare that the research was conducted in the absence of any commercial or financial relationships that could be construed as a potential conflict of interest.

## Footnotes

^1^https://www.who-umc.org/

^2^http://www.ema.europa.eu

^3^https://www.fda.gov/

^4^http://www.mhra.gov.uk

^5^https://www.lareb.nl/en/

^6^https://www.w3.org/RDF/

^7^https://www.w3.org/TR/rdf-schema/

^8^https://www.w3.org/OWL/

^9^Remark: The current paper focuses on the *OpenPVSignal* model per se, and we consider the envisioned software stack facilitating the knowledge graph creation and processing as future work.

^10^https://www.meddra.org/. MedDRA® the Medical Dictionary for Regulatory Activities terminology is the international medical terminology developed under the auspices of the International Council for Harmonisation of Technical Requirements for Pharmaceuticals for Human Use (ICH). The MedDRA® trademark is owned by IFPMA on behalf of ICH.

^11^https://www.nlm.nih.gov/mesh/

^12^https://github.com/OHDSI/CommonDataModel/wiki

^13^https://www.nlm.nih.gov/research/umls/rxnorm/

^14^https://www.nlm.nih.gov/research/umls/sourcereleasedocs/current/NDFRT/

^15^https://www.nlm.nih.gov/research/umls/

^16^https://www.whocc.no/atc/structure_and_principles/

^17^https://www.drugbank.ca/

^18^http://www.who.int/classifications/icd/en/

^19^https://www.snomed.org/snomed-ct

^20^Remark: Concepts contained in WADM are not used to semantically define *OpenPVSignal* concepts. They are only used to create annotations that explicitly declare the original free-text snippet, which is the source of the respective RDF statement in the *OpenPVSignal* instantiation. Therefore, they are not referenced in the *OpenPVSignal* conceptual schema description.

^21^https://github.com/inab-certh/OpenPVSignal/

^22^http://purl.org/OpenPVSignal/OpenPVSignal.owl

^23^http://apps.who.int/medicinedocs/en/d/Js21458en/

^24^https://www.who-umc.org/vigibase/vigibase/

^25^https://www.lareb.nl/en/databank/

^26^https://www.fda.gov/Drugs/DrugSafety/ucm199082.htm

^27^https://github.com/inab-certh/OpenPVSignal/tree/master/examples

^28^https://github.com/FAIRMetrics/Metrics/blob/master/ALL.pdf

^29^http://fairmetrics.org/

^30^Remark: The import of OAE in *OpenPVSignal* increases the size of the model significantly and this leads to vast memory consumption while running reasoners. Removing the OAE import allows a reasoner to build the inferred class hierarchy in seconds. In the presented example instantiations, we used the Hermit reasoner (Hermit, SCR_016006) incorporated in Protégé, in a computer with 16GB RAM and Intel i7 processor.
