# Supplementary material for: OpenPVSignal: Advancing Information Search, Sharing and Reuse on Pharmacovigilance Signals via FAIR Principles and Semantic Web Technologies
Source: Front Pharmacol. 2018 Jun 26;9:609. doi: 10.3389/fphar.2018.00609 (PMC6028717; doi:10.3389/fphar.2018.00609)
Supplement: Supplementary file 1 [file Presentation_1.ZIP › OpenPVSignal.html]

OpenPVSignalOpenPVSignal 

# OpenPVSignal

IRI:
:   http://purl.org/OpenPVSignal/OpenPVSignal.owl

Current version:
:   draft-v0.9-20180129

Imported Ontologies:
:   http://purl.obolibrary.org/obo/oae.owl (visualise it with LODE)
:   http://purl.org/mp (visualise it with LODE)
:   http://www.w3.org/2006/time#2016 (visualise it with LODE)
:   http://www.w3.org/ns/oa# (visualise it with LODE)

Other visualisation:
:   Ontology source

---

## Abstract

OpenPVSignal ontology describes a model able to present pharmacovigilance signal information, originally contained in free-text reports.

## Table of Content

1. Classes
2. Object Properties
3. Data Properties
4. Annotation Properties
5. Namespace Declarations

## Classes

- Adverse Effect
- Adverse Effect Mechanism
- Author
- Bibliographic reference
- Case Report Information
- Clinical Trial Information
- Conclusion
- Condition
- Confidence Interval 0.95
- Disproportionality Analysis Measure
- Dosage
- Drug
- Drug Class
- Drug Class Effect Information
- Drug Exposure Time
- Drug Intake Form
- Drug Usage
- Free text reporting element
- i a o 0000300
- Indication
- Individual Case Safety Report
- Introduction
- Lareb Report
- Literature Information
- Mechanism
- Number of prescriptions on population
- o a e 0000011
- o a e 0000028
- o a e 0000055
- o a e 0000931
- o a e 0001000
- o a e 0001563
- o a e 0001817
- o a e 0001867
- o g m s 0000031
- Patient
- Pharmacovigilance Signal Report
- Proportional Reporting Ratio (PRR)
- Relative Reporting Ratio (RRR)
- Reporting Odds Ratio (ROR)
- Reports group
- Response to Pharmacovigilance Signal Report
- Signal
- Statistical Entity
- Structured Product Labels information
- Summary
- VigiBase report
- Warning Information

### Adverse Effectc back to ToC or Class ToC

**IRI:** http://purl.org/OpenPVSignal/OpenPVSignal.owl#Adverse\_Effect

Noxious and unintended response to a medicinal product.

has super-classes
:   Conditionc

is in domain of
:   has MedDRA code, has MedDRA prefered term

is in range of
:   refers to adverse effectop

### Adverse Effect Mechanismc back to ToC or Class ToC

**IRI:** http://purl.org/OpenPVSignal/OpenPVSignal.owl#Adverse\_Effect\_Mechanism

A free-text description of the mechanism of action related with an adverse effect.

has super-classes
:   Mechanismc

### Authorc back to ToC or Class ToC

**IRI:** http://purl.org/OpenPVSignal/OpenPVSignal.owl#Author

The person or organization that wrote or was responsible for writing the respective PV signal report.

has super-classes
:   Free text reporting elementc
:   agentc
:   organizationc or personc

is in domain of
:   has affiliationdp, has first namedp, has last namedp

is in range of
:   refers to authorop

### Bibliographic referencec back to ToC or Class ToC

**IRI:** http://purl.org/OpenPVSignal/OpenPVSignal.owl#Bibliographic\_reference

Reference to published material that is cited in the PV signal report (e.g. scientific paper, book, report etc.)

has super-classes
:   Free text reporting elementc
:   referencec

### Case Report Informationc back to ToC or Class ToC

**IRI:** http://purl.org/OpenPVSignal/OpenPVSignal.owl#Case\_Report\_Information

Description of the findings from case report publications which are mentioned in the PV signal report.

has super-classes
:   Free text reporting elementc

### Clinical Trial Informationc back to ToC or Class ToC

**IRI:** http://purl.org/OpenPVSignal/OpenPVSignal.owl#Clinical\_trial\_information

Description of the findings from clinical trials which are mentioned in the PV signal report.

has super-classes
:   Free text reporting elementc

### Conclusionc back to ToC or Class ToC

**IRI:** http://purl.org/OpenPVSignal/OpenPVSignal.owl#Conclusion

The free-text description of the main outcome of the PV signal report.

has super-classes
:   Free text reporting elementc

### Conditionc back to ToC or Class ToC

**IRI:** http://purl.org/OpenPVSignal/OpenPVSignal.owl#Condition

A definite pathologic process with a characteristic set of signs and symptoms. It may affect the whole body or any of its parts, and its etiology, pathology, and prognosis may be known or unknown.

has sub-classes
:   Adverse Effectc

### Confidence Interval 0.95c back to ToC or Class ToC

**IRI:** http://purl.org/OpenPVSignal/OpenPVSignal.owl#Confidence\_interval\_0.95

The marginal values of the range in which the respective statistical measure (e.g. PRR or ROR) belongs with a propability of 95%.

has super-classes
:   Statistical Entityc

is in domain of
:   has lower limitdp, has upper limitdp

is in range of
:   refers to confidence intervalop

### Disproportionality Analysis Measurec back to ToC or Class ToC

**IRI:** http://purl.org/OpenPVSignal/OpenPVSignal.owl#Disproportionality\_Analysis\_Measure

Statistical feature depicting the comparison between the rate at which a particular event of interest (e.g. adverse effect) co-occurs with a given drug, and the rate of this event occuring without the drug.

has super-classes
:   Statistical Entityc

has sub-classes
:   Proportional Reporting Ratio (PRR)c, Relative Reporting Ratio (RRR)c, Reporting Odds Ratio (ROR)c

is in domain of
:   refers to number of reportsdp

### Dosagec back to ToC or Class ToC

**IRI:** http://purl.org/OpenPVSignal/OpenPVSignal.owl#Dosage

The regulated administration of individual doses, the quantity of drug to be administered at one time, or the total quantity administered during a specified period, i.e. the dosage not only tells the quantity of medicine to be taken, but it also tells the frequency or the number of times a medicine has to be taken by the patient.

is in domain of
:   refers to dechallenge processdp, refers to dose valuedp, refers to interval between administrationsop, refers to rechallenge processdp

is in range of
:   refers to dosageop

### Drugc back to ToC or Class ToC

**IRI:** http://purl.org/OpenPVSignal/OpenPVSignal.owl#Drug

Product intended to be administered to humans for treating or preventing disease, with the view to making a medical diagnosis or to restore, correct or modify physiological functions

has super-classes
:   o b i 0000047c

is in domain of
:   belongs to classop, has RxNorm code, has drugbank code, has registration datedp, has trade namedp

is in range of
:   refers to drugop

### Drug Classc back to ToC or Class ToC

**IRI:** http://purl.org/OpenPVSignal/OpenPVSignal.owl#DrugClass

A set of medications that have similar chemical structures, the same mechanism of action (i.e., bind to the same biological target), a related mode of action, and/or are used to treat the same disease.

is in range of
:   belongs to classop, refers to classop

### Drug Class Effect Informationc back to ToC or Class ToC

**IRI:** http://purl.org/OpenPVSignal/OpenPVSignal.owl#Drug\_Class\_Effect\_Information

Free text information regarding the effect of the drugs belonging to a specific drug class.

has super-classes
:   Free text reporting elementc

### Drug Exposure Timec back to ToC or Class ToC

**IRI:** http://purl.org/OpenPVSignal/OpenPVSignal.owl#Drug\_Exposure\_Time

The time related information regarding the administration of a drug to a patient (e.g. when it started and when it ended).

is in range of
:   is related with drug exposureop

### Drug Intake Formc back to ToC or Class ToC

**IRI:** http://purl.org/OpenPVSignal/OpenPVSignal.owl#Drug\_Intake\_Form

The form of the drug taken by the patient (e.g. injection, pill etc.).

is in domain of
:   refers to form of intakedp

is in range of
:   is related with drug intake formop

### Drug Usagec back to ToC or Class ToC

**IRI:** http://purl.org/OpenPVSignal/OpenPVSignal.owl#Drug\_Usage

The details of an overall drug administration process (e.g. exposure, intake form, dosage).

is in domain of
:   is related with drug exposureop, is related with drug intake formop, refers to dosageop

is in range of
:   refers to reported drug usageop

### Free text reporting elementc back to ToC or Class ToC

**IRI:** http://purl.org/OpenPVSignal/OpenPVSignal.owl#Free\_text\_reporting\_element

Free-text snippets that could be used in human readable reports (e.g. in printing PDFs)

has super-classes
:   article textc

has sub-classes
:   Authorc, Bibliographic referencec, Case Report Informationc, Clinical Trial Informationc, Conclusionc, Drug Class Effect Informationc, Introductionc, Literature Informationc, Mechanismc, Response to Pharmacovigilance Signal Reportc, Structured Product Labels informationc, Summaryc

is in domain of
:   has contentdp, has subjectdp, is part ofop

is in range of
:   has free text reporting elementop, is part ofop

### i a o 0000300c back to ToC or Class ToC

**IRI:** http://purl.obolibrary.org/obo/IAO\_0000300

is equivalent to
:   Free text reporting elementc

### Indicationc back to ToC or Class ToC

**IRI:** http://purl.org/OpenPVSignal/OpenPVSignal.owl#Indication

A medical condition which makes a particular treatment or procedure advisable

is in range of
:   concerns indication for useop

### Individual Case Safety Reportc back to ToC or Class ToC

**IRI:** http://purl.org/OpenPVSignal/OpenPVSignal.owl#Individual\_Case\_Safety\_Report

A report triggered by a suspicion of a healthcare professional or a patient that observed signs and symptoms could have been caused by a medicine.

has super-classes
:   o a e 0000127c
:   datac

has sub-classes
:   Lareb Reportc, VigiBase reportc

is in domain of
:   belongs to reports groupop, concerns action upon adverse effectdp, has IDdp, has reporter typedp, refers to concomitant drugop, refers to dechallenge outcomedp, refers to interacting drugop, refers to outcome after actiondp, refers to patientop, refers to primary suspect drugop, refers to rechallenge outcomedp, refers to reported drug usageop, refers to secondary suspect drugop, time to onsetop

is in range of
:   is supported by individual case reportop

### Introductionc back to ToC or Class ToC

**IRI:** http://purl.org/OpenPVSignal/OpenPVSignal.owl#Introduction

has super-classes
:   Free text reporting elementc

### Lareb Reportc back to ToC or Class ToC

**IRI:** http://purl.org/OpenPVSignal/OpenPVSignal.owl#Lareb\_Report

An Individual Case Safety Report submitted in the Dutch spontaneous report system maintained by Lareb.

has super-classes
:   Individual Case Safety Reportc

### Literature Informationc back to ToC or Class ToC

**IRI:** http://purl.org/OpenPVSignal/OpenPVSignal.owl#Literature\_information

Description of the findings in scientific literature as contained in the PV signal report.

has super-classes
:   Free text reporting elementc

### Mechanismc back to ToC or Class ToC

**IRI:** http://purl.org/OpenPVSignal/OpenPVSignal.owl#Mechanism

A free-text description of a drug's mechanism of action, as described in the PV signal report.

has super-classes
:   Free text reporting elementc

has sub-classes
:   Adverse Effect Mechanismc

is in range of
:   has mechanismop

### Number of prescriptions on populationc back to ToC or Class ToC

**IRI:** http://purl.org/OpenPVSignal/OpenPVSignal.owl#Number\_of\_prescriptions\_on\_population

The count of the specific drug prescriptions on a population basis.

has super-classes
:   Statistical Entityc

### o a e 0000011c back to ToC or Class ToC

**IRI:** http://purl.obolibrary.org/obo/OAE\_0000011

is equivalent to
:   Drug Usagec

### o a e 0000028c back to ToC or Class ToC

**IRI:** http://purl.obolibrary.org/obo/OAE\_0000028

is equivalent to
:   Signalc

### o a e 0000055c back to ToC or Class ToC

**IRI:** http://purl.obolibrary.org/obo/OAE\_0000055

is equivalent to
:   Warning Informationc

### o a e 0000931c back to ToC or Class ToC

**IRI:** http://purl.obolibrary.org/obo/OAE\_0000931

is equivalent to
:   Drug Exposure Timec

### o a e 0001000c back to ToC or Class ToC

**IRI:** http://purl.obolibrary.org/obo/OAE\_0001000

is equivalent to
:   Adverse Effectc

### o a e 0001563c back to ToC or Class ToC

**IRI:** http://purl.obolibrary.org/obo/OAE\_0001563

is equivalent to
:   Proportional Reporting Ratio (PRR)c

### o a e 0001817c back to ToC or Class ToC

**IRI:** http://purl.obolibrary.org/obo/OAE\_0001817

is equivalent to
:   Patientc

### o a e 0001867c back to ToC or Class ToC

**IRI:** http://purl.obolibrary.org/obo/OAE\_0001867

is equivalent to
:   Dosagec

### o g m s 0000031c back to ToC or Class ToC

**IRI:** http://purl.obolibrary.org/obo/OGMS\_0000031

is equivalent to
:   Conditionc

### Patientc back to ToC or Class ToC

**IRI:** http://purl.org/OpenPVSignal/OpenPVSignal.owl#Patient

Person awaiting or under medical care or treatment by a health professional

has super-classes
:   personc

is in domain of
:   has agedp, has genderdp

is in range of
:   refers to patientop

### Pharmacovigilance Signal Reportc back to ToC or Class ToC

**IRI:** http://purl.org/OpenPVSignal/OpenPVSignal.owl#Pharmacovigilance\_Signal\_Report

Free text reports published by PV monitoring organizations regarding a PV signal. Examples include reports published by FDA, WHO-UMC and Lareb.

has super-classes
:   micropublicationc

is in domain of
:   has free text reporting elementop, has overall conclusiondp, refers to signalop

### Proportional Reporting Ratio (PRR)c back to ToC or Class ToC

**IRI:** http://purl.org/OpenPVSignal/OpenPVSignal.owl#Proportional\_Reporting\_Ratio\_(POR)

Depicts the extent to which a particular adverse effect is reported for individuals taking a specific drug, compared to the frequency at which the same adverse event is reported for patients taking some other drug (or who are taking any drug in a specified class of drugs).

has super-classes
:   Disproportionality Analysis Measurec

### Relative Reporting Ratio (RRR)c back to ToC or Class ToC

**IRI:** http://purl.org/OpenPVSignal/OpenPVSignal.owl#Relative\_Reporting\_Ratio

Depicts the observed frequency of the adverse event to expected frequency in the total population sample.

has super-classes
:   Disproportionality Analysis Measurec

### Reporting Odds Ratio (ROR)c back to ToC or Class ToC

**IRI:** http://purl.org/OpenPVSignal/OpenPVSignal.owl#Reporting\_Odds\_Ratio

The Odds of exposure (to a Medicinal product ) in cases (e.g. individuals with an Adverse reaction) divided by the Odds of exposure in controls (e.g. individuals without the Adverse reaction).

has super-classes
:   Disproportionality Analysis Measurec

### Reports groupc back to ToC or Class ToC

**IRI:** http://purl.org/OpenPVSignal/OpenPVSignal.owl#Reports\_group

A set of Individual Case Safety Reports corresponding to specific criteria (e.g. country of origin).

has super-classes
:   Statistical Entityc

is in domain of
:   has average agedp, has countdp, has median agedp, has min agedp, is subgroup ofop

is in range of
:   belongs to reports groupop, is subgroup ofop

### Response to Pharmacovigilance Signal Reportc back to ToC or Class ToC

**IRI:** http://purl.org/OpenPVSignal/OpenPVSignal.owl#Response\_to\_Pharmacovigilance\_Signal\_Report

Arguments to the claims of a PV signal report, as contained in the PV signal report itself in free-text format. Typically, such a response would be issued by a pharmaceutical company producing the investigated drug.

has super-classes
:   Free text reporting elementc

### Signalc back to ToC or Class ToC

**IRI:** http://purl.org/OpenPVSignal/OpenPVSignal.owl#Signal

Information that arises from one or multiple sources (including observations and experiments), which suggests a new potentially causal association, or a new aspect of a known association, between an intervention and an event or set of related events, either adverse or beneficial, that is judged to be of sufficient likelihood to justify verificatory action.

has super-classes
:   claimc

is in domain of
:   initially identified ondp, is supported by individual case reportop

is in range of
:   refers to signalop

### Statistical Entityc back to ToC or Class ToC

**IRI:** http://purl.org/OpenPVSignal/OpenPVSignal.owl#Statistical\_Entity

Concepts that depict the result of the calculation of specific statistical processes (e.g. disproportionality analysis)

has super-classes
:   datac

has sub-classes
:   Confidence Interval 0.95c, Disproportionality Analysis Measurec, Number of prescriptions on populationc, Reports groupc

is in domain of
:   has valuedp

is in range of
:   is supported by statistical entityop

### Structured Product Labels informationc back to ToC or Class ToC

**IRI:** http://purl.org/OpenPVSignal/OpenPVSignal.owl#Structured\_Product\_Labels\_information

Free-text description of information elaborated the leaflet which is typically part of the drug's packaging (a.k.a Structured Product Label, or Summary of Product Characteristics - SmPC), as contained in the PV signal report.

has super-classes
:   Free text reporting elementc

### Summaryc back to ToC or Class ToC

**IRI:** http://purl.org/OpenPVSignal/OpenPVSignal.owl#Summary

The free text summary of a pharmacovigilance signal report

has super-classes
:   Free text reporting elementc

### VigiBase reportc back to ToC or Class ToC

**IRI:** http://purl.org/OpenPVSignal/OpenPVSignal.owl#VigiBase\_Report

An Individual Case Safety Report stored in the VigiBase database maintained by WHO-UMC.

has super-classes
:   Individual Case Safety Reportc

### Warning Informationc back to ToC or Class ToC

**IRI:** http://purl.org/OpenPVSignal/OpenPVSignal.owl#Warning\_Information

Information related with adverse effects or contraindications that are already identified in various data sources (e.g. product labels).

has super-classes
:   referencec

## Object Properties

- belongs to class
- belongs to reports group
- concerns indication for use
- has free text reporting element
- has mechanism
- is part of
- is related with drug exposure
- is related with drug intake form
- is subgroup of
- is supported by individual case report
- is supported by statistical entity
- refers to adverse effect
- refers to author
- refers to class
- refers to concomitant drug
- refers to confidence interval
- refers to dosage
- refers to drug
- refers to interacting drug
- refers to interval between administrations
- refers to patient
- refers to primary suspect drug
- refers to reported drug usage
- refers to secondary suspect drug
- refers to signal
- time to onset

### belongs to classop back to ToC or Object Property ToC

**IRI:** http://purl.org/OpenPVSignal/OpenPVSignal.owl#hasClass

Relates a drug with the drug class that it belongs to.

has super-properties
:   refers to classop

has domain
:   Drugc

has range
:   Drug Classc

### belongs to reports groupop back to ToC or Object Property ToC

**IRI:** http://purl.org/OpenPVSignal/OpenPVSignal.owl#belongs\_to\_reports\_group

Relates a spontaneous report with a report group.

has domain
:   Individual Case Safety Reportc

has range
:   Reports groupc

### concerns indication for useop back to ToC or Object Property ToC

**IRI:** http://purl.org/OpenPVSignal/OpenPVSignal.owl#concerns\_indication\_for\_use

Identifies the condition which can be considered the reason for the respective drug administration.

has super-properties
:   top object property

has domain
:   Drugc or Drug Classc or Drug Usagec or Reports groupc

has range
:   Indicationc

### has free text reporting elementop back to ToC or Object Property ToC

**IRI:** http://purl.org/OpenPVSignal/OpenPVSignal.owl#has\_free\_text\_reporting\_element

Relates a free-text reporting element with the pharmacovigilance report that it belongs to.

**has characteristics:** inverse functional

has super-properties
:   has elementop

has domain
:   Pharmacovigilance Signal Reportc

has range
:   Free text reporting elementc

### has mechanismop back to ToC or Object Property ToC

**IRI:** http://purl.org/OpenPVSignal/OpenPVSignal.owl#has\_mechanism

Depicts the relation between a drug or a drug class and a mechanism of action.

has domain
:   Drugc or Drug Classc

has range
:   Mechanismc

### is part ofop back to ToC or Object Property ToC

**IRI:** http://purl.org/OpenPVSignal/OpenPVSignal.owl#is\_part\_of

Depicts that a free-text snippet could be part of a larger free-text section.

**has characteristics:** transitive

has domain
:   Free text reporting elementc

has range
:   Free text reporting elementc

### is related with drug exposureop back to ToC or Object Property ToC

**IRI:** http://purl.org/OpenPVSignal/OpenPVSignal.owl#is\_related\_with\_drug\_exposure

Relates the information regarding a specific drug usage with the respective time exposure.

has super-properties
:   top object property

has domain
:   Drug Usagec

has range
:   Drug Exposure Timec

### is related with drug intake formop back to ToC or Object Property ToC

**IRI:** http://purl.org/OpenPVSignal/OpenPVSignal.owl#is\_related\_with\_drug\_intake

Relates the usage of a drug with a specific intake form.

has super-properties
:   top object property

has domain
:   Drug Usagec

has range
:   Drug Intake Formc

### is subgroup ofop back to ToC or Object Property ToC

**IRI:** http://purl.org/OpenPVSignal/OpenPVSignal.owl#is\_subgroup\_of

Enables the identification of a spontaneous report group as part of another spontaneous report group.

has domain
:   Reports groupc

has range
:   Reports groupc

### is supported by individual case reportop back to ToC or Object Property ToC

**IRI:** http://purl.org/OpenPVSignal/OpenPVSignal.owl#is\_supported\_by\_individual\_case\_report

Relates a pharmacovigilance signal with the corresponding individual case reports.

has super-properties
:   supported by dataop

has domain
:   Signalc

has range
:   Individual Case Safety Reportc

### is supported by statistical entityop back to ToC or Object Property ToC

**IRI:** http://purl.org/OpenPVSignal/OpenPVSignal.owl#is\_supported\_by\_statistical\_entity

Relates a pharmacovigilance signal with the supporting statistical data.

**has characteristics:** inverse functional

has super-properties
:   supported by dataop

has sub-properties
:   refers to confidence intervalop

has domain
:   Signalc or Statistical Entityc

has range
:   Statistical Entityc

### refers to adverse effectop back to ToC or Object Property ToC

**IRI:** http://purl.org/OpenPVSignal/OpenPVSignal.owl#refers\_to\_adverse\_effect

**has characteristics:** transitive

has domain
:   Adverse Effectc or Disproportionality Analysis Measurec or Drugc or Individual Case Safety Reportc or Signalc or Warning Informationc

has range
:   Adverse Effectc

### refers to authorop back to ToC or Object Property ToC

**IRI:** http://purl.org/OpenPVSignal/OpenPVSignal.owl#refers\_to\_author

Identifies the author that the respective manuscript refers to.

has super-properties
:   attribution as authorop
:   has attributionop

has domain
:   Pharmacovigilance Signal Reportc or Response to Pharmacovigilance Signal Reportc

has range
:   Authorc

### refers to classop back to ToC or Object Property ToC

**IRI:** http://purl.org/OpenPVSignal/OpenPVSignal.owl#refers\_to\_class

Depicts a reference with the respective drug class.

has sub-properties
:   belongs to classop

has range
:   Drug Classc

### refers to concomitant drugop back to ToC or Object Property ToC

**IRI:** http://purl.org/OpenPVSignal/OpenPVSignal.owl#refers\_to\_concomitant\_drug

Explicitly states that the respective drug is reported as a concomittant drug in the specific spontaneous report.

has super-properties
:   refers to drugop

has domain
:   Individual Case Safety Reportc

### refers to confidence intervalop back to ToC or Object Property ToC

**IRI:** http://purl.org/OpenPVSignal/OpenPVSignal.owl#refers\_to\_confidence\_interval

Depicts a relation with a specific confidence interval.

has super-properties
:   is supported by statistical entityop

has range
:   Confidence Interval 0.95c

### refers to dosageop back to ToC or Object Property ToC

**IRI:** http://purl.org/OpenPVSignal/OpenPVSignal.owl#refers\_to\_dosage

Relates a drug usage with a specifc dosage.

has super-properties
:   top object property

has domain
:   Drug Usagec

has range
:   Dosagec

### refers to drugop back to ToC or Object Property ToC

**IRI:** http://purl.org/OpenPVSignal/OpenPVSignal.owl#refers\_to\_drug

Depicts a relation with the respective drug.

has sub-properties
:   refers to concomitant drugop, refers to interacting drugop, refers to primary suspect drugop, refers to secondary suspect drugop

has domain
:   Disproportionality Analysis Measurec or Dosagec or Drug Classc or Drug Exposure Timec or Drug Intake Formc or Drug Usagec or Individual Case Safety Reportc or Number of prescriptions on populationc or Signalc or Warning Informationc

has range
:   Drugc

### refers to interacting drugop back to ToC or Object Property ToC

**IRI:** http://purl.org/OpenPVSignal/OpenPVSignal.owl#refers\_to\_interacting\_drug

Explicitly states that the respective drug is reported as an interacting drug in the specific spontaneous report.

has super-properties
:   refers to drugop

has domain
:   Individual Case Safety Reportc

### refers to interval between administrationsop back to ToC or Object Property ToC

**IRI:** http://purl.org/OpenPVSignal/OpenPVSignal.owl#refers\_to\_interval\_between\_administrations

Identifies the time passed between two consecutive drug administrations.

has domain
:   Dosagec

has range
:   duration description

### refers to patientop back to ToC or Object Property ToC

**IRI:** http://purl.org/OpenPVSignal/OpenPVSignal.owl#refers\_to\_patient

Identifies the patient that the respective spontaneous report refers to.

**has characteristics:** functional

has super-properties
:   attributed toop

has domain
:   Individual Case Safety Reportc

has range
:   Patientc

### refers to primary suspect drugop back to ToC or Object Property ToC

**IRI:** http://purl.org/OpenPVSignal/OpenPVSignal.owl#refers\_to\_primary\_suspect\_drug

Explicitly states that the respective drug is reported as the primary suspect drug in the specific spontaneous report.

**has characteristics:** functional

has super-properties
:   refers to drugop

has domain
:   Individual Case Safety Reportc

### refers to reported drug usageop back to ToC or Object Property ToC

**IRI:** http://purl.org/OpenPVSignal/OpenPVSignal.owl#refers\_to\_reported\_drug\_usage

Relates a spontaneous report with the respective drug usages.

**has characteristics:** functional

has domain
:   Individual Case Safety Reportc

has range
:   Drug Usagec

### refers to secondary suspect drugop back to ToC or Object Property ToC

**IRI:** http://purl.org/OpenPVSignal/OpenPVSignal.owl#refers\_to\_secondary\_suspect\_drug

Explicitly states that the respective drug is reported as a secondary suspect drug in the specific spontaneous report.

has super-properties
:   refers to drugop

has domain
:   Individual Case Safety Reportc

### refers to signalop back to ToC or Object Property ToC

**IRI:** http://purl.org/OpenPVSignal/OpenPVSignal.owl#refers\_to\_signal

Relates a pharmacovigilance signal report with the respective signal.

has super-properties
:   arguesop

has domain
:   Pharmacovigilance Signal Reportc

has range
:   Signalc

### time to onsetop back to ToC or Object Property ToC

**IRI:** http://purl.org/OpenPVSignal/OpenPVSignal.owl#time\_to\_onset

Identifies the time passed before the adverse effect symptoms start, according to the respective spontaneous report.

**has characteristics:** functional

has domain
:   Individual Case Safety Reportc

has range
:   duration description

## Data Properties

- concerns action upon adverse effect
- has affiliation
- has age
- has average age
- has content
- has count
- has count of men
- has count of women
- has creation date
- has first name
- has gender
- has ID
- has last name
- has lower limit
- has max age
- has median age
- has min age
- has overall conclusion
- has registration date
- has reporter type
- has subject
- has trade name
- has upper limit
- has value
- initially identified on
- modified on
- refers to database
- refers to dechallenge outcome
- refers to dechallenge process
- refers to dose value
- refers to form of intake
- refers to number of reports
- refers to outcome after action
- refers to rechallenge outcome
- refers to rechallenge process

### concerns action upon adverse effectdp back to ToC or Data Property ToC

**IRI:** http://purl.org/OpenPVSignal/OpenPVSignal.owl#concerns\_action\_upon\_adverse\_effect

Depicts the actions upon the diagnosis of the adverse effect, as reported in the respective spontaneous report.

has domain
:   Individual Case Safety Reportc

has range
:   { "discontinued" , "hospitalization" , "no change" }

### has affiliationdp back to ToC or Data Property ToC

**IRI:** http://purl.org/OpenPVSignal/OpenPVSignal.owl#has\_affiliation

Depicts the respective author's professional affiliation(s).

has domain
:   Authorc

has range
:   literal

### has agedp back to ToC or Data Property ToC

**IRI:** http://purl.org/OpenPVSignal/OpenPVSignal.owl#has\_age

Refers to the patient's age. It should be noted that the target value can be either an integer or an enumeration depiction the respective age decade.

**has characteristics:** functional

has domain
:   Patientc

has range
:   integer or ({ "1-10" , "11-20" , "21-30" , "31-40" , "41-50" , "51-60" , "61-70" , "71 or older" })

### has average agedp back to ToC or Data Property ToC

**IRI:** http://purl.org/OpenPVSignal/OpenPVSignal.owl#has\_average\_age

Depicts the average age of the patients referred by a specific spontaneous reports group.

has domain
:   Reports groupc

has range
:   float or integer

### has contentdp back to ToC or Data Property ToC

**IRI:** http://purl.org/OpenPVSignal/OpenPVSignal.owl#has\_content

Links to the free text of the respective element. Formatting information could be included (e.g. in HTML format).

has domain
:   Free text reporting elementc

has range
:   literal

### has countdp back to ToC or Data Property ToC

**IRI:** http://purl.org/OpenPVSignal/OpenPVSignal.owl#has\_count

Depicts the total number of patients belonging to a specific report group.

has sub-properties
:   has count of mendp, has count of womendp

has domain
:   Reports groupc

has range
:   integer

### has count of mendp back to ToC or Data Property ToC

**IRI:** http://purl.org/OpenPVSignal/OpenPVSignal.owl#has\_count\_of\_men

Depicts the total number of men belonging to a specific report group.

has super-properties
:   has countdp

### has count of womendp back to ToC or Data Property ToC

**IRI:** http://purl.org/OpenPVSignal/OpenPVSignal.owl#has\_count\_of\_women

Depicts the total number of women belonging to a specific report group.

has super-properties
:   has countdp

### has creation datedp back to ToC or Data Property ToC

**IRI:** http://purl.org/OpenPVSignal/OpenPVSignal.owl#has\_creation\_date

Declares the date(s) that the respective pharmacovigilance signal report has been created.

**has characteristics:** functional

has super-properties
:   top data property

has domain
:   Disproportionality Analysis Measurec or Individual Case Safety Reportc or Pharmacovigilance Signal Reportc or Response to Pharmacovigilance Signal Reportc

has range
:   date

### has first namedp back to ToC or Data Property ToC

**IRI:** http://purl.org/OpenPVSignal/OpenPVSignal.owl#has\_first\_name

Declares the person's first name.

has domain
:   Authorc

has range
:   literal

### has genderdp back to ToC or Data Property ToC

**IRI:** http://purl.org/OpenPVSignal/OpenPVSignal.owl#has\_gender

Depicts the patient's gender.

**has characteristics:** functional

has domain
:   Patientc

has range
:   { "female" , "male" }

### has IDdp back to ToC or Data Property ToC

**IRI:** http://purl.org/OpenPVSignal/OpenPVSignal.owl#has\_ID

Refers to the ID used for the specific spontaneous report, to be referenced in the overall signal report.

**has characteristics:** functional

has domain
:   Individual Case Safety Reportc

has range
:   literal

### has last namedp back to ToC or Data Property ToC

**IRI:** http://purl.org/OpenPVSignal/OpenPVSignal.owl#has\_last\_name

Declares the person's last name.

has super-properties
:   top data property

has domain
:   Authorc

has range
:   literal

### has lower limitdp back to ToC or Data Property ToC

**IRI:** http://purl.org/OpenPVSignal/OpenPVSignal.owl#has\_lower\_limit

Depicts the confidence interval's lower limit.

has domain
:   Confidence Interval 0.95c

has range
:   decimal

### has max agedp back to ToC or Data Property ToC

**IRI:** http://purl.org/OpenPVSignal/OpenPVSignal.owl#has\_max\_age

Depicts the maximum age of the patients referred by a specific spontaneous reports group.

has range
:   float or integer

### has median agedp back to ToC or Data Property ToC

**IRI:** http://purl.org/OpenPVSignal/OpenPVSignal.owl#has\_median\_age

Depicts the median age of the patients referred by a specific spontaneous reports group.

has domain
:   Reports groupc

has range
:   float or integer

### has min agedp back to ToC or Data Property ToC

**IRI:** http://purl.org/OpenPVSignal/OpenPVSignal.owl#has\_min\_age

Depicts the minimum age of the patients referred by a specific spontaneous reports group.

has domain
:   Reports groupc

has range
:   float or integer

### has overall conclusiondp back to ToC or Data Property ToC

**IRI:** http://purl.org/OpenPVSignal/OpenPVSignal.owl#has\_overall\_conclusion

It depicts the final conclusion of the report in an enumeratable form.

has domain
:   Pharmacovigilance Signal Reportc

has range
:   { "Further investigation needed" , "causal association" , "causal association most probable" , "causal association probable" , "no clear conclusion" , "rejected" }

### has registration datedp back to ToC or Data Property ToC

**IRI:** http://purl.org/OpenPVSignal/OpenPVSignal.owl#has\_registration\_date

Declares the date(s) that the respective drug has been registered.

has domain
:   Drugc

has range
:   date

### has reporter typedp back to ToC or Data Property ToC

**IRI:** http://purl.org/OpenPVSignal/OpenPVSignal.owl#has\_reporter\_type

Depicts the category of the person that submitted the respective spontaneous report.

has domain
:   Individual Case Safety Reportc

has range
:   { "clinical trial report" , "health care professional, other than physician" , "informal carer" , "other" , "patient" , "pharmaceutical company" , "pharmacist" , "physician" , "study" }

### has subjectdp back to ToC or Data Property ToC

**IRI:** http://purl.org/OpenPVSignal/OpenPVSignal.owl#has\_subject

Depicts the subject of the respective free-text section.

has domain
:   Free text reporting elementc

has range
:   literal

### has trade namedp back to ToC or Data Property ToC

**IRI:** http://purl.org/OpenPVSignal/OpenPVSignal.owl#has\_trade\_name

Depicts the respective drug's trade name.

has domain
:   Drugc

has range
:   literal

### has upper limitdp back to ToC or Data Property ToC

**IRI:** http://purl.org/OpenPVSignal/OpenPVSignal.owl#has\_upper\_limit

Depicts the confidence interval's upper limit.

has domain
:   Confidence Interval 0.95c

has range
:   decimal

### has valuedp back to ToC or Data Property ToC

**IRI:** http://purl.org/OpenPVSignal/OpenPVSignal.owl#has\_value

Depicts the value of the respective statistical data item.

has super-properties
:   valuedp
:   valuedp

has domain
:   Statistical Entityc

has range
:   decimal

### initially identified ondp back to ToC or Data Property ToC

**IRI:** http://purl.org/OpenPVSignal/OpenPVSignal.owl#initially\_identified\_on

Depicts the date that the respective signal has been originally identified on.

has domain
:   Signalc

has range
:   date or date time or g year or g year month

### modified ondp back to ToC or Data Property ToC

**IRI:** http://purl.org/OpenPVSignal/OpenPVSignal.owl#modified\_on

Declares the date(s) that the respective pharmacovigilance signal report has been modified.

**has characteristics:** functional

has super-properties
:   edited ondp

has domain
:   Pharmacovigilance Signal Reportc or Response to Pharmacovigilance Signal Reportc

has range
:   date time

### refers to databasedp back to ToC or Data Property ToC

**IRI:** http://purl.org/OpenPVSignal/OpenPVSignal.owl#refers\_to\_database

Identifies the database that the respective data are related with.

has super-properties
:   published ondp

has domain
:   Disproportionality Analysis Measurec or Reports groupc

has range
:   { "Eudravigilance" , "FAERS" , "LAREB" , "WHO Vigibase" }

### refers to dechallenge outcomedp back to ToC or Data Property ToC

**IRI:** http://purl.org/OpenPVSignal/OpenPVSignal.owl#refers\_to\_dechallenge\_outcome

Depicts the outcome of the dechallenge process applied to the respective adverse effect, as reported in the spontaneous report.

has domain
:   Individual Case Safety Reportc

has range
:   { "negative - condition still applied appeared" , "positive - condition not applied" , "unknown" }

### refers to dechallenge processdp back to ToC or Data Property ToC

**IRI:** http://purl.org/OpenPVSignal/OpenPVSignal.owl#refers\_to\_dechallenge\_process

Depicts if the respective dosage is used as part of a dechallenge process.

has domain
:   Dosagec

has range
:   boolean

### refers to dose valuedp back to ToC or Data Property ToC

**IRI:** http://purl.org/OpenPVSignal/OpenPVSignal.owl#refers\_to\_dose\_value

Depicts the dose value of the respective dosage scheme, including the units (e.g. mg).

has domain
:   Dosagec

has range
:   literal

### refers to form of intakedp back to ToC or Data Property ToC

**IRI:** http://purl.org/OpenPVSignal/OpenPVSignal.owl#refers\_to\_form\_of\_intake

Depicts the intake form of a respective drug.

**has characteristics:** functional

has domain
:   Drug Intake Formc

has range
:   { "collyrium" , "inhaled" , "injected" , "injected (intramuscular)" , "injected (intraveneous)" , "nasal" , "oral" , "other" , "rectal" , "smoked" , "subcutaneous" , "sublingual" , "topical (skin)" }

### refers to number of reportsdp back to ToC or Data Property ToC

**IRI:** http://purl.org/OpenPVSignal/OpenPVSignal.owl#refers\_to\_number\_of\_reports

Identifies the count of the spontaneous reports based on which the respective statistical data item has been calculated.

has domain
:   Disproportionality Analysis Measurec

has range
:   int

### refers to outcome after actiondp back to ToC or Data Property ToC

**IRI:** http://purl.org/OpenPVSignal/OpenPVSignal.owl#refers\_to\_outcome\_after\_action

Depicts the outcome of the action against the respective adverse effect, as reported in the spontaneous report.

**has characteristics:** functional

has domain
:   Individual Case Safety Reportc

has range
:   { "death" , "hospitalization for treatment" , "no recovery" , "permanent injury" , "recovery after drug withdrawal" , "recovery after hospitalization" , "recovery with no further information" , "recovery without drug withdrawal" , "unknown" }

### refers to rechallenge outcomedp back to ToC or Data Property ToC

**IRI:** http://purl.org/OpenPVSignal/OpenPVSignal.owl#refers\_to\_rechallenge\_outcome

Depicts the outcome of the rechallenge process applied to the respective adverse effect, as reported in the spontaneous report.

has super-properties
:   top data property

has domain
:   Individual Case Safety Reportc

has range
:   { "death" , "hospitalization for treatment" , "negative rechallenge - condition not appeared" , "permanent injury" , "recovery after drug withdrawl" , "recovery after hospitalization" , "recovery with no further information" , "recovery without drug withdrawl" , "unknown" }

### refers to rechallenge processdp back to ToC or Data Property ToC

**IRI:** http://purl.org/OpenPVSignal/OpenPVSignal.owl#refers\_to\_rechallenge\_process

Depicts if the respective dosage is used as part of a rechallenge process.

has domain
:   Dosagec

has range
:   boolean

## Annotation Properties

- has ATC code
- has broad synonym
- has db xref
- has drugbank code
- has exact synonym
- has ICD code
- has MedDRA code
- has MedDRA prefered term
- has MeSH tree number
- has RxNorm code
- has SNOMED-CT code
- has target
- i a o 0000119
- title

### has ATC codeap back to ToC or Annotation Property ToC

**IRI:** http://purl.org/OpenPVSignal/OpenPVSignal.owl#has\_ATC\_code

Identifies the ATC code of the respective drug.

has super-properties
:   has db xrefap

has range
:   string

### has broad synonymap back to ToC or Annotation Property ToC

**IRI:** http://www.geneontology.org/formats/oboInOwl#hasBroadSynonym

### has db xrefap back to ToC or Annotation Property ToC

**IRI:** http://www.geneontology.org/formats/oboInOwl#hasDbXref

has sub-properties
:   has ATC codeap, has ICD codeap, has MeSH tree numberap, has MedDRA codeap, has MedDRA prefered termap, has RxNorm codeap, has SNOMED-CT codeap, has drugbank codeap

### has drugbank codeap back to ToC or Annotation Property ToC

**IRI:** http://purl.org/OpenPVSignal/OpenPVSignal.owl#has\_drugbank\_code

Identifies the drugbank code of the respective drug.

has super-properties
:   has db xrefap

has domain
:   Drugc

has range
:   string

### has exact synonymap back to ToC or Annotation Property ToC

**IRI:** http://www.geneontology.org/formats/oboInOwl#hasExactSynonym

### has ICD codeap back to ToC or Annotation Property ToC

**IRI:** http://purl.org/OpenPVSignal/OpenPVSignal.owl#has\_ICD\_code

Identifies the ICD code of the respective condition.

has super-properties
:   has db xrefap

has range
:   string

### has MedDRA codeap back to ToC or Annotation Property ToC

**IRI:** http://purl.org/OpenPVSignal/OpenPVSignal.owl#has\_MedDRA\_code

Identifies the MedDRA code of the respective adverse effect.

has super-properties
:   has db xrefap

has domain
:   Adverse Effectc

has range
:   int

### has MedDRA prefered termap back to ToC or Annotation Property ToC

**IRI:** http://purl.org/OpenPVSignal/OpenPVSignal.owl#has\_MedDRA\_prefered\_term

Identifies the MedDRA preferred term regarding the respective adverse effect.

has super-properties
:   has db xrefap

has domain
:   Adverse Effectc

has range
:   string

### has MeSH tree numberap back to ToC or Annotation Property ToC

**IRI:** http://purl.org/OpenPVSignal/OpenPVSignal.owl#has\_MeSH\_tree\_number

Identifies the MeSH tree code of the respective entity.

has super-properties
:   has db xrefap

has range
:   string

### has RxNorm codeap back to ToC or Annotation Property ToC

**IRI:** http://purl.org/OpenPVSignal/OpenPVSignal.owl#has\_RxNorm\_code

Identifies the RxNorm code of the respective drug.

has super-properties
:   has db xrefap

has domain
:   Drugc

has range
:   string

### has SNOMED-CT codeap back to ToC or Annotation Property ToC

**IRI:** http://purl.org/OpenPVSignal/OpenPVSignal.owl#has\_SNOMED-CT\_code

Indentifies the SNOMED-CT code of the respective entity

has super-properties
:   has db xrefap

has range
:   string

### has targetap back to ToC or Annotation Property ToC

**IRI:** http://www.w3.org/ns/oa#hasTarget

### i a o 0000119ap back to ToC or Annotation Property ToC

**IRI:** http://purl.obolibrary.org/obo/IAO\_0000119

### titleap back to ToC or Annotation Property ToC

**IRI:** http://purl.org/dc/elements/1.1/title

## Namespace Declarations back to ToC

*default namespace*
:   http://purl.org/OpenPVSignal/OpenPVSignal.owl#

OpenPVSignal
:   http://purl.org/OpenPVSignal/OpenPVSignal.owl#

dc
:   http://purl.org/dc/elements/1.1/

mp
:   http://purl.org/mp/

oa
:   http://www.w3.org/ns/oa#

obo
:   http://purl.obolibrary.org/obo/

oboInOwl
:   http://www.geneontology.org/formats/oboInOwl#

openpvsignal
:   http://purl.org/OpenPVSignal/

owl
:   http://www.w3.org/2002/07/owl#

prov
:   http://www.w3.org/ns/prov#

purl-org
:   http://purl.org/

rdf
:   http://www.w3.org/1999/02/22-rdf-syntax-ns#

rdfs
:   http://www.w3.org/2000/01/rdf-schema#

time
:   http://www.w3.org/2006/time#

xsd
:   http://www.w3.org/2001/XMLSchema#

This HTML document was obtained by processing the OWL ontology source code through LODE, *Live OWL Documentation Environment*, developed by Silvio Peroni.
