# Supplementary material for: OpenPVSignal: Advancing Information Search, Sharing and Reuse on Pharmacovigilance Signals via FAIR Principles and Semantic Web Technologies
Source: Front Pharmacol. 2018 Jun 26;9:609. doi: 10.3389/fphar.2018.00609 (PMC6028717; doi:10.3389/fphar.2018.00609)
Supplement: Supplementary file 1 [file Presentation_1.ZIP › OpenPVSignal.pdf]

OpenPVSignal

IRI:
http://purl.org/OpenPVSignal/OpenPVSignal.owl

Current version:
draft-v0.9-20180129

Imported Ontologies:
http://purl.obolibrary.org/obo/oae.owl (visualise it with LODE)
http://purl.org/mp (visualise it with LODE)
http://www.w3.org/2006/time#2016 (visualise it with LODE)
http://www.w3.org/ns/oa# (visualise it with LODE)

Other visualisation:
Ontology source

Abstract

OpenPVSignal ontology describes a model able to present pharmacovigilance signal information, originally contained in free-text reports.

Table of Content

1. Classes
2. Object Properties
3. Data Properties
4. Annotation Properties
5. Namespace Declarations

Classes

Adverse Effect   Adverse Effect Mechanism   Author   Bibliographic reference
Case Report Information   Clinical Trial Information   Conclusion   Condition
Confidence Interval 0.95   Disproportionality Analysis Measure   Dosage   Drug
Drug Class   Drug Class Effect Information   Drug Exposure Time
Drug Intake Form   Drug Usage   Free text reporting element   i a o 0000300
Indication   Individual Case Safety Report   Introduction   Lareb Report
Literature Information   Mechanism   Number of prescriptions on population
o a e 0000011   o a e 0000028   o a e 0000055   o a e 0000931   o a e 0001000
o a e 0001563   o a e 0001817   o a e 0001867   o g m s 0000031   Patient

Pharmacovigilance Signal Report   Proportional Reporting Ratio (PRR)
Relative Reporting Ratio (RRR)   Reporting Odds Ratio (ROR)   Reports group
Response to Pharmacovigilance Signal Report   Signal   Statistical Entity
Structured Product Labels information   Summary   VigiBase report
Warning Information

Adverse Effect[c]

back to ToC or Class ToC

IRI: http://purl.org/OpenPVSignal/OpenPVSignal.owl#Adverse_Effect

Noxious and unintended response to a medicinal product.

has super-classes

Condition[c]

is in domain of

has MedDRA code, has MedDRA prefered term

is in range of

refers to adverse effect[op]

Adverse Effect Mechanism[c]

back to ToC or Class ToC

IRI: http://purl.org/OpenPVSignal/OpenPVSignal.owl#Adverse_Effect_Mechanism

A free-text description of the mechanism of action related with an adverse effect.

has super-classes

Mechanism[c]

Author[c]

back to ToC or Class ToC

IRI: http://purl.org/OpenPVSignal/OpenPVSignal.owl#Author

The person or organization that wrote or was responsible for writing the respective PV signal report.

has super-classes

Free text reporting element[c]

agent[c]

organization[c] **or** person[c]

is in domain of

has affiliation[dp], has first name[dp], has last name[dp]

is in range of

refers to author[op]

Bibliographic reference[c]

back to ToC or Class ToC

IRI: http://purl.org/OpenPVSignal/OpenPVSignal.owl#Bibliographic_reference

Reference to published material that is cited in the PV signal report (e.g. scientific paper, book, report etc.)

has super-classes

Free text reporting element[c]
reference[c]

Case Report Information[c]

back to ToC or Class ToC

IRI: http://purl.org/OpenPVSignal/OpenPVSignal.owl#Case_Report_Information

Description of the findings from case report publications which are mentioned in the PV signal report.

has super-classes

Free text reporting element[c]

Clinical Trial Information[c]

back to ToC or Class ToC

IRI: http://purl.org/OpenPVSignal/OpenPVSignal.owl#Clinical_trial_information

Description of the findings from clinical trials which are mentioned in the PV signal report.

has super-classes

Free text reporting element[c]

Conclusion[c]

back to ToC or Class ToC

IRI: http://purl.org/OpenPVSignal/OpenPVSignal.owl#Conclusion

The free-text description of the main outcome of the PV signal report.

has super-classes

Free text reporting element[c]

Condition[c]

back to ToC or Class ToC

IRI: http://purl.org/OpenPVSignal/OpenPVSignal.owl#Condition

A definite pathologic process with a characteristic set of signs and symptoms. It may affect the whole body or any of its parts, and its etiology, pathology, and prognosis may be known or unknown.

has sub-classes

Adverse Effect[c]

Confidence Interval 0.95[c]

back to ToC or Class ToC

IRI: http://purl.org/OpenPVSignal/OpenPVSignal.owl#Confidence_interval_0.95

The marginal values of the range in which the respective statistical measure (e.g. PRR or ROR) belongs with a propability of 95%.

has super-classes

Statistical Entity[c]

is in domain of

has lower limit[dp], has upper limit[dp]

is in range of

refers to confidence interval[op]

Disproportionality Analysis Measure[c]

back to ToC or Class ToC

IRI: http://purl.org/OpenPVSignal/OpenPVSignal.owl#Disproportionality_Analysis_Measure

Statistical feature depicting the comparison between the rate at which a particular event of interest (e.g. adverse effect) co-occurs with a given drug, and the rate of this event occuring without the drug.

has super-classes

Statistical Entity[c]

has sub-classes

Proportional Reporting Ratio (PRR)[c], Relative Reporting Ratio (RRR)[c], Reporting Odds Ratio (ROR)[c]

is in domain of

refers to number of reports[dp]

Dosage[c]

back to ToC or Class ToC

IRI: http://purl.org/OpenPVSignal/OpenPVSignal.owl#Dosage

The regulated administration of individual doses, the quantity of drug to be administered at one time, or the total quantity administered during a specified period, i.e. the dosage

not only tells the quantity of medicine to be taken, but it also tells the frequency or the number of times a medicine has to be taken by the patient.

is in domain of

refers to dechallenge process[dp], refers to dose value[dp], refers to interval between administrations[op], refers to rechallenge process[dp]

is in range of

refers to dosage[op]

Drug[c]

back to ToC or Class ToC

IRI: http://purl.org/OpenPVSignal/OpenPVSignal.owl#Drug

Product intended to be administered to humans for treating or preventing disease, with the view to making a medical diagnosis or to restore, correct or modify physiological functions

has super-classes

o b i 0000047[c]

is in domain of

belongs to class[op], has RxNorm code, has drugbank code, has registration date[dp], has trade name[dp]

is in range of

refers to drug[op]

Drug Class[c]

back to ToC or Class ToC

IRI: http://purl.org/OpenPVSignal/OpenPVSignal.owl#DrugClass

A set of medications that have similar chemical structures, the same mechanism of action (i.e., bind to the same biological target), a related mode of action, and/or are used to treat the same disease.

is in range of

belongs to class[op], refers to class[op]

Drug Class Effect Information[c]

back to ToC or Class ToC

IRI: http://purl.org/OpenPVSignal/OpenPVSignal.owl#Drug_Class_Effect_Information

Free text information regarding the effect of the drugs belonging to a specific drug class.

has super-classes

Free text reporting element[c]

Drug Exposure Time[c]

back to ToC or Class ToC

IRI: http://purl.org/OpenPVSignal/OpenPVSignal.owl#Drug_Exposure_Time

The time related information regarding the administration of a drug to a patient (e.g. when it started and when it ended).

is in range of

is related with drug exposure[op]

Drug Intake Form[c]

back to ToC or Class ToC

IRI: http://purl.org/OpenPVSignal/OpenPVSignal.owl#Drug_Intake_Form

The form of the drug taken by the patient (e.g. injection, pill etc.).

is in domain of

refers to form of intake[dp]

is in range of

is related with drug intake form[op]

Drug Usage[c]

back to ToC or Class ToC

IRI: http://purl.org/OpenPVSignal/OpenPVSignal.owl#Drug_Usage

The details of an overall drug administration process (e.g. exposure, intake form, dosage).

is in domain of

is related with drug exposure[op], is related with drug intake form[op], refers to dosage[op]

is in range of

refers to reported drug usage[op]

Free text reporting element[c]

back to ToC or Class ToC

IRI: http://purl.org/OpenPVSignal/OpenPVSignal.owl#Free_text_reporting_element

Free-text snippets that could be used in human readable reports (e.g. in printing PDFs)

has super-classes

article text[c]

has sub-classes

Author[c], Bibliographic reference[c], Case Report Information[c], Clinical Trial Information[c], Conclusion[c], Drug Class Effect Information[c], Introduction[c], Literature Information[c], Mechanism[c], Response to Pharmacovigilance Signal Report[c], Structured Product Labels information[c], Summary[c]

is in domain of

has content[dp], has subject[dp], is part of[op]

is in range of

has free text reporting element[op], is part of[op]

i a o 0000300[c]

back to ToC or Class ToC

IRI: http://purl.obolibrary.org/obo/IAO_0000300

is equivalent to

Free text reporting element[c]

Indication[c]

back to ToC or Class ToC

IRI: http://purl.org/OpenPVSignal/OpenPVSignal.owl#Indication

A medical condition which makes a particular treatment or procedure advisable

is in range of

concerns indication for use[op]

Individual Case Safety Report[c]

back to ToC or Class ToC

IRI: http://purl.org/OpenPVSignal/OpenPVSignal.owl#Individual_Case_Safety_Report

A report triggered by a suspicion of a healthcare professional or a patient that observed signs and symptoms could have been caused by a medicine.

has super-classes

o a e 0000127[c]

data[c]

has sub-classes

Lareb Report[c], VigiBase report[c]

is in domain of

belongs to reports group[op], concerns action upon adverse effect[dp], has ID[dp], has reporter type[dp], refers to concomitant drug[op], refers to dechallenge outcome[dp], refers to interacting drug[op], refers to outcome after action[dp], refers to patient[op], refers to primary suspect drug[op], refers to rechallenge outcome[dp], refers to reported drug usage[op], refers to secondary suspect drug[op], time to onset[op]

is in range of

is supported by individual case report[op]

Introduction[c]

back to ToC or Class ToC

IRI: http://purl.org/OpenPVSignal/OpenPVSignal.owl#Introduction

has super-classes

Free text reporting element[c]

Lareb Report[c]

back to ToC or Class ToC

IRI: http://purl.org/OpenPVSignal/OpenPVSignal.owl#Lareb_Report

An Individual Case Safety Report submitted in the Dutch spontaneous report system maintained by Lareb.

has super-classes

Individual Case Safety Report[c]

Literature Information[c]

back to ToC or Class ToC

IRI: http://purl.org/OpenPVSignal/OpenPVSignal.owl#Literature_information

Description of the findings in scientific literature as contained in the PV signal report.

has super-classes

Free text reporting element[c]

Mechanism[c]

back to ToC or Class ToC

IRI: http://purl.org/OpenPVSignal/OpenPVSignal.owl#Mechanism

A free-text description of a drug's mechanism of action, as described in the PV signal

report.

has super-classes

Free text reporting element[c]

has sub-classes

Adverse Effect Mechanism[c]

is in range of

has mechanism[op]

Number of prescriptions on population[c]

back to ToC or Class ToC

IRI: http://purl.org/OpenPVSignal/OpenPVSignal.owl#Number_of_prescriptions_on_population

The count of the specific drug prescriptions on a population basis.

has super-classes

Statistical Entity[c]

o a e 0000011[c]

back to ToC or Class ToC

IRI: http://purl.obolibrary.org/obo/OAE_0000011

is equivalent to

Drug Usage[c]

o a e 0000028[c]

back to ToC or Class ToC

IRI: http://purl.obolibrary.org/obo/OAE_0000028

is equivalent to

Signal[c]

o a e 0000055[c]

back to ToC or Class ToC

IRI: http://purl.obolibrary.org/obo/OAE_0000055

is equivalent to

Warning Information[c]

o a e 0000931[c]

back to ToC or Class ToC

IRI: http://purl.obolibrary.org/obo/OAE_0000931

is equivalent to

Drug Exposure Time[c]

o a e 0001000[c]

back to ToC or Class ToC

IRI: http://purl.obolibrary.org/obo/OAE_0001000

is equivalent to

Adverse Effect[c]

o a e 0001563[c]

back to ToC or Class ToC

IRI: http://purl.obolibrary.org/obo/OAE_0001563

is equivalent to

Proportional Reporting Ratio (PRR)[c]

o a e 0001817[c]

back to ToC or Class ToC

IRI: http://purl.obolibrary.org/obo/OAE_0001817

is equivalent to

Patient[c]

o a e 0001867[c]

back to ToC or Class ToC

IRI: http://purl.obolibrary.org/obo/OAE_0001867

is equivalent to

Dosage[c]

o g m s 0000031[c]

back to ToC or Class ToC

IRI: http://purl.obolibrary.org/obo/OGMS_0000031

is equivalent to

Condition[c]

Patient[c]

back to ToC or Class ToC

IRI: http://purl.org/OpenPVSignal/OpenPVSignal.owl#Patient

Person awaiting or under medical care or treatment by a health professional

has super-classes

person[c]

is in domain of

has age[dp], has gender[dp]

is in range of

refers to patient[op]

Pharmacovigilance Signal Report[c]

back to ToC or Class ToC

IRI: http://purl.org/OpenPVSignal/OpenPVSignal.owl#Pharmacovigilance_Signal_Report

Free text reports published by PV monitoring organizations regarding a PV signal. Examples include reports published by FDA, WHO-UMC and Lareb.

has super-classes

micropublication[c]

is in domain of

has free text reporting element[op], has overall conclusion[dp], refers to signal[op]

Proportional Reporting Ratio (PRR)[c]

back to ToC or Class ToC

IRI: http://purl.org/OpenPVSignal/OpenPVSignal.owl#Proportional_Reporting_Ratio_(POR)

Depicts the extent to which a particular adverse effect is reported for individuals taking a specific drug, compared to the frequency at which the same adverse event is reported for patients taking some other drug (or who are taking any drug in a specified class of drugs).

has super-classes

Disproportionality Analysis Measure[c]

Relative Reporting Ratio (RRR)[c]

back to ToC or Class ToC

IRI: http://purl.org/OpenPVSignal/OpenPVSignal.owl#Relative_Reporting_Ratio

Depicts the observed frequency of the adverse event to expected frequency in the total population sample.

has super-classes

Disproportionality Analysis Measure[c]

Reporting Odds Ratio (ROR)[c]

back to ToC or Class ToC

IRI: http://purl.org/OpenPVSignal/OpenPVSignal.owl#Reporting_Odds_Ratio

The Odds of exposure (to a Medicinal product) in cases (e.g. individuals with an Adverse reaction) divided by the Odds of exposure in controls (e.g. individuals without the Adverse reaction).

has super-classes

Disproportionality Analysis Measure[c]

Reports group[c]

back to ToC or Class ToC

IRI: http://purl.org/OpenPVSignal/OpenPVSignal.owl#Reports_group

A set of Individual Case Safety Reports corresponding to specific criteria (e.g. country of origin).

has super-classes

Statistical Entity[c]

is in domain of

has average age[dp], has count[dp], has median age[dp], has min age[dp], is subgroup of[op]

is in range of

belongs to reports group[op], is subgroup of[op]

Response to Pharmacovigilance Signal Report[c]

back to ToC or Class ToC

IRI: http://purl.org/OpenPVSignal/OpenPVSignal.owl#Response_to_Pharmacovigilance_Signal_Report

Arguments to the claims of a PV signal report, as contained in the PV signal report itself in free-text format. Typically, such a response would be issued by a pharmaceutical company producing the investigated drug.

has super-classes

Free text reporting element[c]

Signal[c]

back to ToC or Class ToC

IRI: http://purl.org/OpenPVSignal/OpenPVSignal.owl#Signal

Information that arises from one or multiple sources (including observations and experiments), which suggests a new potentially causal association, or a new aspect of a known association, between an intervention and an event or set of related events, either adverse or beneficial, that is judged to be of sufficient likelihood to justify verificatory action.

has super-classes

claim[c]

is in domain of

initially identified on[dp], is supported by individual case report[op]

is in range of

refers to signal[op]

Statistical Entity[c]

back to ToC or Class ToC

IRI: http://purl.org/OpenPVSignal/OpenPVSignal.owl#Statistical_Entity

Concepts that depict the result of the calculation of specific statistical processes (e.g. disproportionality analysis)

has super-classes

data[c]

has sub-classes

Confidence Interval 0.95[c], Disproportionality Analysis Measure[c], Number of prescriptions on population[c], Reports group[c]

is in domain of

has value[dp]

is in range of

is supported by statistical entity[op]

Structured Product Labels information[c]

back to ToC or Class ToC

IRI: http://purl.org/OpenPVSignal/OpenPVSignal.owl#Structured_Product_Labels_information

Free-text description of information elaborated the leaflet which is typically part of the drug's packaging (a.k.a Structured Product Label, or Summary of Product Characteristics - SmPC), as contained in the PV signal report.

has super-classes

Free text reporting element[c]

Summary[c]

back to ToC or Class ToC

IRI: http://purl.org/OpenPVSignal/OpenPVSignal.owl#Summary

The free text summary of a pharmacovigilance signal report

has super-classes

Free text reporting element[c]

VigiBase report[c]

back to ToC or Class ToC

IRI: http://purl.org/OpenPVSignal/OpenPVSignal.owl#VigiBase_Report

An Individual Case Safety Report stored in the VigiBase database maintained by WHO-UMC.

has super-classes

Individual Case Safety Report[c]

Warning Information[c]

back to ToC or Class ToC

IRI: http://purl.org/OpenPVSignal/OpenPVSignal.owl#Warning_Information

Information related with adverse effects or contraindications that are already identified in various data sources (e.g. product labels).

has super-classes

reference[c]

Object Properties

belongs to class belongs to reports group concerns indication for use
has free text reporting element has mechanism is part of
is related with drug exposure is related with drug intake form is subgroup of
is supported by individual case report is supported by statistical entity
refers to adverse effect refers to author refers to class
refers to concomitant drug refers to confidence interval refers to dosage
refers to drug refers to interacting drug refers to interval between administrations
refers to patient refers to primary suspect drug refers to reported drug usage
refers to secondary suspect drug refers to signal time to onset

belongs to class[op]

back to ToC or Object Property ToC

IRI: http://purl.org/OpenPVSignal/OpenPVSignal.owl#hasClass

Relates a drug with the drug class that it belongs to.

has super-properties
refers to class[op]
has domain
Drug[c]
has range
Drug Class[c]

belongs to reports group[op]

back to ToC or Object Property ToC

IRI: http://purl.org/OpenPVSignal/OpenPVSignal.owl#belongs_to_reports_group

Relates a spontaneous report with a report group.

has domain
Individual Case Safety Report[c]
has range
Reports group[c]

concerns indication for use[op]

back to ToC or Object Property ToC

IRI: http://purl.org/OpenPVSignal/OpenPVSignal.owl#concerns_indication_for_use

Identifies the condition which can be considered the reason for the respective drug administration.

has super-properties
top object property
has domain
Drug[c] **or** Drug Class[c] **or** Drug Usage[c] **or** Reports group[c]
has range
Indication[c]

has free text reporting element[op]

back to ToC or Object Property ToC

IRI: http://purl.org/OpenPVSignal/OpenPVSignal.owl#has_free_text_reporting_element

Relates a free-text reporting element with the pharmacovigilance report that it belongs to.

has characteristics: inverse functional

has super-properties

has element[op]

has domain

Pharmacovigilance Signal Report[c]

has range

Free text reporting element[c]

has mechanism[op]

back to ToC or Object Property ToC

IRI: http://purl.org/OpenPVSignal/OpenPVSignal.owl#has_mechanism

Depicts the relation between a drug or a drug class and a mechanism of action.

has domain

Drug[c] **or** Drug Class[c]

has range

Mechanism[c]

is part of[op]

back to ToC or Object Property ToC

IRI: http://purl.org/OpenPVSignal/OpenPVSignal.owl#is_part_of

Depicts that a free-text snippet could be part of a larger free-text section.

has characteristics: transitive

has domain

Free text reporting element[c]

has range

Free text reporting element[c]

is related with drug exposure[op]

back to ToC or Object Property ToC

IRI: http://purl.org/OpenPVSignal/OpenPVSignal.owl#is_related_with_drug_exposure

Relates the information regarding a specific drug usage with the respective time exposure.

has super-properties

top object property

has domain

Drug Usage[c]

has range

Drug Exposure Time[c]

is related with drug intake form[op]

back to ToC or Object Property ToC

IRI: http://purl.org/OpenPVSignal/OpenPVSignal.owl#is_related_with_drug_intake

Relates the usage of a drug with a specific intake form.

has super-properties
: top object property

has domain
: Drug Usage[c]

has range
: Drug Intake Form[c]

is subgroup of[op]

back to ToC or Object Property ToC

IRI: http://purl.org/OpenPVSignal/OpenPVSignal.owl#is_subgroup_of

Enables the identification of a spontaneous report group as part of another spontaneous report group.

has domain
: Reports group[c]

has range
: Reports group[c]

is supported by individual case report[op]

back to ToC or Object Property ToC

IRI: http://purl.org/OpenPVSignal/OpenPVSignal.owl#is_supported_by_individual_case_report

Relates a pharmacovigilance signal with the corresponding individual case reports.

has super-properties
: supported by data[op]

has domain
: Signal[c]

has range
: Individual Case Safety Report[c]

is supported by statistical entity[op]

back to ToC or Object Property ToC

IRI: http://purl.org/OpenPVSignal/OpenPVSignal.owl#is_supported_by_statistical_entity

Relates a pharmacovigilance signal with the supporting statistical data.

has characteristics: inverse functional

has super-properties

supported by data[op]

has sub-properties

refers to confidence interval[op]

has domain

Signal[c] **or** Statistical Entity[c]

has range

Statistical Entity[c]

refers to adverse effect[op]

back to ToC or Object Property ToC

IRI: http://purl.org/OpenPVSignal/OpenPVSignal.owl#refers_to_adverse_effect

has characteristics: transitive

has domain

Adverse Effect[c] **or** Disproportionality Analysis Measure[c] **or** Drug[c] **or** Individual Case Safety Report[c] **or** Signal[c] **or** Warning Information[c]

has range

Adverse Effect[c]

refers to author[op]

back to ToC or Object Property ToC

IRI: http://purl.org/OpenPVSignal/OpenPVSignal.owl#refers_to_author

Identifies the author that the respective manuscript refers to.

has super-properties

attribution as author[op]

has attribution[op]

has domain

Pharmacovigilance Signal Report[c] **or** Response to Pharmacovigilance Signal Report[c]

has range

Author[c]

refers to class^op

back to ToC or Object Property ToC

IRI: http://purl.org/OpenPVSignal/OpenPVSignal.owl#refers_to_class

Depicts a reference with the respective drug class.

has sub-properties

belongs to class^op

has range

Drug Class^c

refers to concomitant drug^op

back to ToC or Object Property ToC

IRI: http://purl.org/OpenPVSignal/OpenPVSignal.owl#refers_to_concomitant_drug

Explicitly states that the respective drug is reported as a concomittant drug in the specific spontaneous report.

has super-properties

refers to drug^op

has domain

Individual Case Safety Report^c

refers to confidence interval^op

back to ToC or Object Property ToC

IRI: http://purl.org/OpenPVSignal/OpenPVSignal.owl#refers_to_confidence_interval

Depicts a relation with a specific confidence interval.

has super-properties

is supported by statistical entity^op

has range

Confidence Interval 0.95^c

refers to dosage^op

back to ToC or Object Property ToC

IRI: http://purl.org/OpenPVSignal/OpenPVSignal.owl#refers_to_dosage

Relates a drug usage with a specifc dosage.

has super-properties

top object property

has domain

Drug Usage[c]

has range

Dosage[c]

refers to drug[op]

back to ToC or Object Property ToC

IRI: http://purl.org/OpenPVSignal/OpenPVSignal.owl#refers_to_drug

Depicts a relation with the respective drug.

has sub-properties

refers to concomitant drug[op], refers to interacting drug[op], refers to primary suspect drug[op], refers to secondary suspect drug[op]

has domain

Disproportionality Analysis Measure[c] **or** Dosage[c] **or** Drug Class[c] **or** Drug Exposure Time[c] **or** Drug Intake Form[c] **or** Drug Usage[c] **or** Individual Case Safety Report[c] **or** Number of prescriptions on population[c] **or** Signal[c] **or** Warning Information[c]

has range

Drug[c]

refers to interacting drug[op]

back to ToC or Object Property ToC

IRI: http://purl.org/OpenPVSignal/OpenPVSignal.owl#refers_to_interacting_drug

Explicitly states that the respective drug is reported as an interacting drug in the specific spontaneous report.

has super-properties

refers to drug[op]

has domain

Individual Case Safety Report[c]

refers to interval between administrations[op]

back to ToC or Object Property ToC

IRI: http://purl.org/OpenPVSignal/OpenPVSignal.owl#refers_to_interval_between_administrations

Identifies the time passed between two consecutive drug administrations.

has domain

Dosage[c]

has range

duration description

refers to patient^op

back to ToC or Object Property ToC

IRI: http://purl.org/OpenPVSignal/OpenPVSignal.owl#refers_to_patient

Identifies the patient that the respective spontaneous report refers to.

has characteristics: functional

has super-properties

attributed to^op

has domain

Individual Case Safety Report^c

has range

Patient^c

refers to primary suspect drug^op

back to ToC or Object Property ToC

IRI: http://purl.org/OpenPVSignal/OpenPVSignal.owl#refers_to_primary_suspect_drug

Explicitly states that the respective drug is reported as the primary suspect drug in the specific spontaneous report.

has characteristics: functional

has super-properties

refers to drug^op

has domain

Individual Case Safety Report^c

refers to reported drug usage^op

back to ToC or Object Property ToC

IRI: http://purl.org/OpenPVSignal/OpenPVSignal.owl#refers_to_reported_drug_usage

Relates a spontaneous report with the respective drug usages.

has characteristics: functional

has domain

Individual Case Safety Report^c

has range

Drug Usage^c

refers to secondary suspect drug^op

back to ToC or Object Property ToC

IRI: http://purl.org/OpenPVSignal/OpenPVSignal.owl#refers_to_secondary_suspect_drug

Explicitly states that the respective drug is reported as a secondary suspect drug in the specific spontaneous report.

has super-properties

refers to drug^op

has domain

Individual Case Safety Report^c

refers to signal^op

back to ToC or Object Property ToC

IRI: http://purl.org/OpenPVSignal/OpenPVSignal.owl#refers_to_signal

Relates a pharmacovigilance signal report with the respective signal.

has super-properties

argues^op

has domain

Pharmacovigilance Signal Report^c

has range

Signal^c

time to onset^op

back to ToC or Object Property ToC

IRI: http://purl.org/OpenPVSignal/OpenPVSignal.owl#time_to_onset

Identifies the time passed before the adverse effect symptoms start, according to the respective spontaneous report.

has characteristics: functional

has domain

Individual Case Safety Report[c]

has range

duration description

Data Properties

concerns action upon adverse effect has affiliation has age has average age has content has count has count of men has count of women has creation date has first name has gender has ID has last name has lower limit has max age has median age has min age has overall conclusion has registration date has reporter type has subject has trade name has upper limit has value initially identified on modified on refers to database refers to dechallenge outcome refers to dechallenge process refers to dose value refers to form of intake refers to number of reports refers to outcome after action refers to rechallenge outcome refers to rechallenge process

concerns action upon adverse effect[dp]

back to ToC or Data Property ToC

IRI: http://purl.org/OpenPVSignal/OpenPVSignal.owl#concerns_action_upon_adverse_effect

Depicts the actions upon the diagnosis of the adverse effect, as reported in the respective spontaneous report.

has domain

Individual Case Safety Report[c]

has range

{ "discontinued" , "hospitalization" , "no change" }

has affiliation[dp]

back to ToC or Data Property ToC

IRI: http://purl.org/OpenPVSignal/OpenPVSignal.owl#has_affiliation

Depicts the respective author's professional affiliation(s).

has domain

Author[c]

has range

literal

has age[dp]

back to ToC or Data Property ToC

IRI: http://purl.org/OpenPVSignal/OpenPVSignal.owl#has_age

Refers to the patient's age. It should be noted that the target value can be either an integer or an enumeration depiction the respective age decade.

has characteristics: functional

has domain

Patient[c]

has range

integer **or** ({ "1-10" , "11-20" , "21-30" , "31-40" , "41-50" , "51-60" , "61-70" , "71 or older" })

has average age[dp]

back to ToC or Data Property ToC

IRI: http://purl.org/OpenPVSignal/OpenPVSignal.owl#has_average_age

Depicts the average age of the patients referred by a specific spontaneous reports group.

has domain

Reports group[c]

has range

float **or** integer

has content[dp]

back to ToC or Data Property ToC

IRI: http://purl.org/OpenPVSignal/OpenPVSignal.owl#has_content

Links to the free text of the respective element. Formatting information could be included (e.g. in HTML format).

has domain

Free text reporting element[c]

has range

literal

has count[dp]

back to ToC or Data Property ToC

IRI: http://purl.org/OpenPVSignal/OpenPVSignal.owl#has_count

Depicts the total number of patients belonging to a specific report group.

has sub-properties

has count of men[dp], has count of women[dp]

has domain

Reports group[c]

has range

integer

has count of men[dp]

back to ToC or Data Property ToC

IRI: http://purl.org/OpenPVSignal/OpenPVSignal.owl#has_count_of_men

Depicts the total number of men belonging to a specific report group.

has super-properties

has count[dp]

has count of women[dp]

back to ToC or Data Property ToC

IRI: http://purl.org/OpenPVSignal/OpenPVSignal.owl#has_count_of_women

Depicts the total number of women belonging to a specific report group.

has super-properties

has count[dp]

has creation date[dp]

back to ToC or Data Property ToC

IRI: http://purl.org/OpenPVSignal/OpenPVSignal.owl#has_creation_date

Declares the date(s) that the respective pharmacovigilance signal report has been created.

has characteristics: functional

has super-properties

top data property

has domain

Disproportionality Analysis Measure^c **or** Individual Case Safety Report^c **or** Pharmacovigilance Signal Report^c **or** Response to Pharmacovigilance Signal Report^c

has range

date

has first name^dp

back to ToC or Data Property ToC

IRI: http://purl.org/OpenPVSignal/OpenPVSignal.owl#has_first_name

Declares the person's first name.

has domain

Author^c

has range

literal

has gender^dp

back to ToC or Data Property ToC

IRI: http://purl.org/OpenPVSignal/OpenPVSignal.owl#has_gender

Depicts the patient's gender.

has characteristics: functional

has domain

Patient^c

has range

{ "female" , "male" }

has ID^dp

back to ToC or Data Property ToC

IRI: http://purl.org/OpenPVSignal/OpenPVSignal.owl#has_ID

Refers to the ID used for the specific spontaneous report, to be referenced in the overall signal report.

has characteristics: functional

has domain

Individual Case Safety Report^c

has range

literal

has last name[dp]

back to ToC or Data Property ToC

IRI: http://purl.org/OpenPVSignal/OpenPVSignal.owl#has_last_name

Declares the person's last name.

has super-properties
top data property
has domain
Author[c]
has range
literal

has lower limit[dp]

back to ToC or Data Property ToC

IRI: http://purl.org/OpenPVSignal/OpenPVSignal.owl#has_lower_limit

Depicts the confidence interval's lower limit.

has domain
Confidence Interval 0.95[c]
has range
decimal

has max age[dp]

back to ToC or Data Property ToC

IRI: http://purl.org/OpenPVSignal/OpenPVSignal.owl#has_max_age

Depicts the maximum age of the patients referred by a specific spontaneous reports group.

has range
float **or** integer

has median age[dp]

back to ToC or Data Property ToC

IRI: http://purl.org/OpenPVSignal/OpenPVSignal.owl#has_median_age

Depicts the median age of the patients referred by a specific spontaneous reports group.

has domain
Reports group[c]
has range

float **or** integer

has min age[dp]

back to ToC or Data Property ToC

IRI: http://purl.org/OpenPVSignal/OpenPVSignal.owl#has_min_age

Depicts the minimum age of the patients referred by a specific spontaneous reports group.

has domain

Reports group[c]

has range

float **or** integer

has overall conclusion[dp]

back to ToC or Data Property ToC

IRI: http://purl.org/OpenPVSignal/OpenPVSignal.owl#has_overall_conclusion

It depicts the final conclusion of the report in an enumeratable form.

has domain

Pharmacovigilance Signal Report[c]

has range

{ "Further investigation needed" , "causal association" , "causal association most probable" , "causal association probable" , "no clear conclusion" , "rejected" }

has registration date[dp]

back to ToC or Data Property ToC

IRI: http://purl.org/OpenPVSignal/OpenPVSignal.owl#has_registration_date

Declares the date(s) that the respective drug has been registered.

has domain

Drug[c]

has range

date

has reporter type[dp]

back to ToC or Data Property ToC

IRI: http://purl.org/OpenPVSignal/OpenPVSignal.owl#has_reporter_type

Depicts the category of the person that submitted the respective spontaneous report.

has domain

Individual Case Safety Report[c]

has range

{ "clinical trial report" , "health care professional, other than physician" , "informal carer" , "other" , "patient" , "pharmaceutical company" , "pharmacist" , "physician" , "study" }

has subject[dp]

back to ToC or Data Property ToC

IRI: http://purl.org/OpenPVSignal/OpenPVSignal.owl#has_subject

Depicts the subject of the respective free-text section.

has domain

Free text reporting element[c]

has range

literal

has trade name[dp]

back to ToC or Data Property ToC

IRI: http://purl.org/OpenPVSignal/OpenPVSignal.owl#has_trade_name

Depicts the respective drug's trade name.

has domain

Drug[c]

has range

literal

has upper limit[dp]

back to ToC or Data Property ToC

IRI: http://purl.org/OpenPVSignal/OpenPVSignal.owl#has_upper_limit

Depicts the confidence interval's upper limit.

has domain

Confidence Interval 0.95[c]

has range

decimal

has value[dp]

back to ToC or Data Property ToC

IRI: http://purl.org/OpenPVSignal/OpenPVSignal.owl#has_value

Depicts the value of the respective statistical data item.

has super-properties

value[dp]

value[dp]

has domain

Statistical Entity[c]

has range

decimal

initially identified on[dp]

back to ToC or Data Property ToC

IRI: http://purl.org/OpenPVSignal/OpenPVSignal.owl#initially_identified_on

Depicts the date that the respective signal has been originally identified on.

has domain

Signal[c]

has range

date **or** date time **or** g year **or** g year month

modified on[dp]

back to ToC or Data Property ToC

IRI: http://purl.org/OpenPVSignal/OpenPVSignal.owl#modified_on

Declares the date(s) that the respective pharmacovigilance signal report has been modified.

has characteristics: functional

has super-properties

edited on[dp]

has domain

Pharmacovigilance Signal Report[c] **or** Response to Pharmacovigilance Signal Report[c]

has range

date time

refers to database[dp]

back to ToC or Data Property ToC

IRI: http://purl.org/OpenPVSignal/OpenPVSignal.owl#refers_to_database

Identifies the database that the respective data are related with.

has super-properties

published on[dp]

has domain

Disproportionality Analysis Measure[c] **or** Reports group[c]

has range

{ "Eudravigilance" , "FAERS" , "LAREB" , "WHO Vigibase" }

refers to dechallenge outcome[dp]

back to ToC or Data Property ToC

IRI: http://purl.org/OpenPVSignal/OpenPVSignal.owl#refers_to_dechallenge_outcome

Depicts the outcome of the dechallenge process applied to the respective adverse effect, as reported in the spontaneous report.

has domain

Individual Case Safety Report[c]

has range

{ "negative - condition still applied appeared" , "positive - condition not applied" , "unknown" }

refers to dechallenge process[dp]

back to ToC or Data Property ToC

IRI: http://purl.org/OpenPVSignal/OpenPVSignal.owl#refers_to_dechallenge_process

Depicts if the respective dosage is used as part of a dechallenge process.

has domain

Dosage[c]

has range

boolean

refers to dose value[dp]

back to ToC or Data Property ToC

IRI: http://purl.org/OpenPVSignal/OpenPVSignal.owl#refers_to_dose_value

Depicts the dose value of the respective dosage scheme, including the units (e.g. mg).

has domain

Dosage[c]

has range

literal

refers to form of intake[dp]

back to ToC or Data Property ToC

IRI: http://purl.org/OpenPVSignal/OpenPVSignal.owl#refers_to_form_of_intake

Depicts the intake form of a respective drug.

has characteristics: functional

has domain

Drug Intake Form[c]

has range

{ "collyrium" , "inhaled" , "injected" , "injected (intramuscular)" , "injected (intraveneous)" , "nasal" , "oral" , "other" , "rectal" , "smoked" , "subcutaneous" , "sublingual" , "topical (skin)" }

refers to number of reports[dp]

back to ToC or Data Property ToC

IRI: http://purl.org/OpenPVSignal/OpenPVSignal.owl#refers_to_number_of_reports

Identifies the count of the spontaneous reports based on which the respective statistical data item has been calculated.

has domain

Disproportionality Analysis Measure[c]

has range

int

refers to outcome after action[dp]

back to ToC or Data Property ToC

IRI: http://purl.org/OpenPVSignal/OpenPVSignal.owl#refers_to_outcome_after_action

Depicts the outcome of the action against the respective adverse effect, as reported in the spontaneous report.

has characteristics: functional

has domain

Individual Case Safety Report[c]

has range

{ "death" , "hospitalization for treatment" , "no recovery" , "permanent injury" , "recovery after drug withdrawal" , "recovery after hospitalization" , "recovery with no further information" , "recovery without drug withdrawal" , "unknown" }

refers to rechallenge outcome[dp]

back to ToC or Data Property ToC

IRI: http://purl.org/OpenPVSignal/OpenPVSignal.owl#refers_to_rechallenge_outcome

Depicts the outcome of the rechallenge process applied to the respective adverse effect, as reported in the spontaneous report.

has super-properties
top data property

has domain
Individual Case Safety Report[c]

has range
{ "death" , "hospitalization for treatment" , "negative rechallenge - condition not appeared" , "permanent injury" , "recovery after drug withdrawl" , "recovery after hospitalization" , "recovery with no further information" , "recovery without drug withdrawl" , "unknown" }

refers to rechallenge process[dp]

back to ToC or Data Property ToC

IRI: http://purl.org/OpenPVSignal/OpenPVSignal.owl#refers_to_rechallenge_process

Depicts if the respective dosage is used as part of a rechallenge process.

has domain
Dosage[c]

has range
boolean

Annotation Properties

has ATC code has broad synonym has db xref has drugbank code
has exact synonym has ICD code has MedDRA code
has MedDRA prefered term has MeSH tree number has RxNorm code
has SNOMED-CT code has target i a o 0000119 title

has ATC code[ap]

back to ToC or Annotation Property ToC

IRI: http://purl.org/OpenPVSignal/OpenPVSignal.owl#has_ATC_code

Identifies the ATC code of the respective drug.

has super-properties
has db xref[ap]

has range
string

has broad synonym[ap]

back to ToC or Annotation Property ToC

IRI: http://www.geneontology.org/formats/oboInOwl#hasBroadSynonym

has db xref[ap]

back to ToC or Annotation Property ToC

IRI: http://www.geneontology.org/formats/oboInOwl#hasDbXref

has sub-properties

has ATC code[ap], has ICD code[ap], has MeSH tree number[ap], has MedDRA code[ap], has MedDRA prefered term[ap], has RxNorm code[ap], has SNOMED-CT code[ap], has drugbank code[ap]

has drugbank code[ap]

back to ToC or Annotation Property ToC

IRI: http://purl.org/OpenPVSignal/OpenPVSignal.owl#has_drugbank_code

Identifies the drugbank code of the respective drug.

has super-properties

has db xref[ap]

has domain

Drug[c]

has range

string

has exact synonym[ap]

back to ToC or Annotation Property ToC

IRI: http://www.geneontology.org/formats/oboInOwl#hasExactSynonym

has ICD code[ap]

back to ToC or Annotation Property ToC

IRI: http://purl.org/OpenPVSignal/OpenPVSignal.owl#has_ICD_code

Identifies the ICD code of the respective condition.

has super-properties

has db xref[ap]

has range

string

has MedDRA code[ap]

back to ToC or Annotation Property ToC

IRI: http://purl.org/OpenPVSignal/OpenPVSignal.owl#has_MedDRA_code

Identifies the MedDRA code of the respective adverse effect.

has super-properties
has db xref[ap]
has domain
Adverse Effect[c]
has range
int

has MedDRA prefered term[ap]

back to ToC or Annotation Property ToC

IRI: http://purl.org/OpenPVSignal/OpenPVSignal.owl#has_MedDRA_prefered_term

Identifies the MedDRA preferred term regarding the respective adverse effect.

has super-properties
has db xref[ap]
has domain
Adverse Effect[c]
has range
string

has MeSH tree number[ap]

back to ToC or Annotation Property ToC

IRI: http://purl.org/OpenPVSignal/OpenPVSignal.owl#has_MeSH_tree_number

Identifies the MeSH tree code of the respective entity.

has super-properties
has db xref[ap]
has range
string

has RxNorm code[ap]

back to ToC or Annotation Property ToC

IRI: http://purl.org/OpenPVSignal/OpenPVSignal.owl#has_RxNorm_code

Identifies the RxNorm code of the respective drug.

has super-properties
: has db xref[ap]

has domain
: Drug[c]

has range
: string

has SNOMED-CT code[ap]

back to ToC or Annotation Property ToC

IRI: http://purl.org/OpenPVSignal/OpenPVSignal.owl#has_SNOMED-CT_code

Indentifies the SNOMED-CT code of the respective entity

has super-properties
: has db xref[ap]

has range
: string

has target[ap]

back to ToC or Annotation Property ToC

IRI: http://www.w3.org/ns/oa#hasTarget

i a o 0000119[ap]

back to ToC or Annotation Property ToC

IRI: http://purl.obolibrary.org/obo/IAO_0000119

title[ap]

back to ToC or Annotation Property ToC

IRI: http://purl.org/dc/elements/1.1/title

Namespace Declarations

back to ToC

default namespace
: http://purl.org/OpenPVSignal/OpenPVSignal.owl#

OpenPVSignal
: http://purl.org/OpenPVSignal/OpenPVSignal.owl#

dc
: http://purl.org/dc/elements/1.1/

mp

http://purl.org/mp/

oa

http://www.w3.org/ns/oa#

obo

http://purl.obolibrary.org/obo/

oboInOwl

http://www.geneontology.org/formats/oboInOwl#

openpvsignal

http://purl.org/OpenPVSignal/

owl

http://www.w3.org/2002/07/owl#

prov

http://www.w3.org/ns/prov#

purl-org

http://purl.org/

rdf

http://www.w3.org/1999/02/22-rdf-syntax-ns#

rdfs

http://www.w3.org/2000/01/rdf-schema#

time

http://www.w3.org/2006/time#

xsd

http://www.w3.org/2001/XMLSchema#

This HTML document was obtained by processing the OWL ontology source code through LODE, *Live OWL Documentation Environment*, developed by Silvio Peroni.
